# Amino Acid Nutrition in Poultry: A Review

**DOI:** 10.3390/ani15223323

**Published:** 2025-11-18

**Authors:** Taiwo Alabi, Sunday Adedokun

**Affiliations:** Department of Animal and Food Sciences, University of Kentucky, Lexington, KY 40546, USA; tjal231@uky.edu

**Keywords:** amino acid, feed efficiency, precision nutrition, SIAAD

## Abstract

Amino acids (AA) play a crucial role in poultry production, directly influencing feed efficiency, growth, immune function, and environmental impact. Over the years, advancements in feed formulation, digestibility assessments, and precision nutrition have contributed to improving poultry performance through AA nutrition while reducing nitrogen (N) excretion. Standardized ileal amino acid digestibility (SIAAD) has become a key tool for accurately evaluating AA bioavailability, moving beyond less precise estimate of apparent ileal digestibility (AID), to ensure that dietary formulations precisely meet the specific needs of poultry at different production stages, while correcting for basal ileal endogenous amino acid losses (BIEAAL) and providing more accurate, additive values for feed formulation. Additionally, innovations in synthetic amino acids (SAA), exogenous enzyme supplementation, and alternative protein sources have further enhanced AA utilization, offering sustainable solutions for modern poultry farming. However, challenges remain in optimizing AA metabolism, minimizing N excretion, and refining feeding strategies to optimize efficiency. This review explores the physiological significance of AAs in poultry, discusses factors affecting their digestibility and utilization, and examines emerging technologies for improving AA nutrition. Understanding these aspects is essential in advancing poultry production while ensuring sustainability and economic viability.

## Simple Summary

Amino acids are vital nutrients in poultry production, supporting growth, egg and meat yield, feed efficiency, and overall health. Modern poultry diets focus on digestible amino acids rather than crude protein, which allows for more precise nutrition and reduced nitrogen waste. Standardized ileal amino acid digestibility (SIAAD) provides accurate estimates for diet formulation, while strategies such as enzyme supplementation and alternative protein sources further enhance amino acid utilization. This review highlights recent advances in amino acid nutrition and their role in supporting efficient, sustainable, and environmentally responsible poultry production.

## 1. Introduction

The poultry industry is a vital contributor to global animal protein production, supplying both meat and eggs as essential sources of high-quality nutrients [1]. Globally, poultry meat accounts for approximately 40% of total meat consumption, while eggs serve as a significant source of dietary protein [1,2]. Consequently, optimizing poultry nutrition is crucial for sustaining a stable food ecosystem, and amino acid (AA) nutrition is fundamental in this optimization. Amino acids, which are the building blocks of proteins, play pivotal roles in poultry nutrition by influencing growth, reproduction, and overall health [3,4]. They are categorized into dietary essential and non-essential AAs, each with distinct functions and dietary requirements.

Beyond their structural role in protein synthesis, AAs contribute to various physiological processes essential for poultry performance. Dietary supplementation with key essential AAs such as methionine, tryptophan, lysine, threonine, glycine, and proline has been associated with improved growth performance, egg quality, and immune function [5,6]. Tryptophan, for example, supports neuroendocrine function, modulates stress responses, and enhances immune organ development in broilers [7]. Under heat stress, AA metabolism is disrupted, and interventions such as oral L-citrulline supplementation have been shown to enhance thermotolerance in laying hens [8]. These targeted responses underscore the need for precision nutrition, particularly under challenging environmental conditions, to sustain productivity and minimize N excretion in poultry production [9,10].

Precision formulation of AA in a diet has emerged as a key strategy for improving environmental sustainability in poultry production. Empirical findings indicate that dietary crude protein (CP) reductions, when precisely balanced with crystalline AAs, can effectively reduce N excretion without compromising birds’ performance [9,11]. This approach not only improves N utilization but also contributes to a reduced environmental footprint associated with feed production and manure management [12,13]. Furthermore, precision feeding strategies that balance AA supply with bird requirements have been shown to improve feed efficiency and reduce N output, thereby enhancing both performance and environmental outcomes [12,14]. Failure to balance AA profiles precisely may not only impair performance but also lead to excessive N excretion and avoidable high feed cost [15,16].

Recent advancements in feed technology have focused on improving AA digestibility, metabolism, and utilization within biological systems [17]. Ileal digestibility, particularly standardized ileal amino acid digestibility (SIAAD), is now the preferred method for estimating AA bioavailability in poultry and swine, as this provides more accurate values for feed formulation [18,19]. However, the presence of anti-nutritional compounds and the impact of feed processing can reduce AA absorption, necessitating the development of more efficient delivery strategies [20,21]. These include the use of chelated AAs and purified crystalline forms aimed at enhancing utilization under varying production conditions [22,23].

This review synthesizes recent advances in AA nutrition in poultry, with emphasis on digestibility systems, precise dietary formulation, and the environmental and economic outcomes of optimized AA use. It covers key areas such as AA digestibility, SIAAD, AA delivery innovations, and strategies to reduce N excretion without compromising performance. By integrating data-driven findings, this review provides evidence-based approaches for improving feed efficiency, sustaining production under challenging conditions, and minimizing environmental impact.

## 2. Essential Versus Non-Essential Amino Acids: Definitions and Roles

### 2.1. Essential Amino Acids (EAAs)

Essential AAs (indispensable AAs) are those that the body cannot synthesize in sufficient quantities and must be supplied through the diet. These include lysine, methionine, tryptophan, threonine, isoleucine, arginine, leucine, histidine, phenylalanine, and valine. Table 1 summarizes the classification and function of these AAs. Notably, isoleucine, valine, and leucine are branched-chain amino acids (BCAAs) [24], which make up 20% of AAs in animal and plant proteins [25]. Some AAs are termed conditionally essential because, although they can be synthesized endogenously, their synthesis may be insufficient under specific physiological or developmental conditions, thus necessitating dietary supplementation. Glycine is often regarded as conditionally essential in young poultry [10,26] due to their limited capacity for endogenous synthesis. Similarly, cysteine and tyrosine are considered conditionally EAAs in poultry because they can be synthesized endogenously from methionine and phenylalanine, respectively, via the trans-sulfuration and hydroxylation pathways [27,28,29]. Among the EAAs, lysine, methionine, valine, threonine, and tryptophan are typically the most limiting in conventional poultry feed formulations [26,30,31]. However, the order of limiting AAs may vary with alternative feedstuffs. For instance, canola meal-based diets tend to be first limited by lysine [32], while wheat-soybean meal (wheat-SBM) diets are often deficient in lysine and threonine [33]. Fernandez et al. [34] reported that methionine is the first limiting AA in corn-SBM (C-SBM). However, among the EAAs, methionine and cysteine are classified as sulfur-containing AAs, and they play a vital role in methyl group donation, antioxidative defense, and feather development [35,36,37]. In addition, according to Bunchasak [38], methionine acts as a precursor for cysteine. It is involved in the methylation process, while cysteine, a precursor to glutathione, acts as an antioxidant to scavenge reactive oxygen species [28,39,40].

### 2.2. Non-Essential Amino Acids (NEAAs)

The non-essential AAs are synthesized endogenously and are critical for metabolic regulation, tissue development, and overall performance in poultry [1,25,70]. These include glycine, serine, proline, tyrosine, alanine, glutamine, glutamate, aspartate, asparagine, and cysteine. Glycine, serine, glutamine, and proline play significant physiological roles that extend beyond protein synthesis [71]. For instance, glycine supports collagen formation, used as a feed additive, and acts as a potent antioxidant in poultry diets [67,72,73]. Recent findings suggest that supplementing arginine to the broiler diet improves growth performance and enhances immune response [74,75]. In addition, proline and glutamine are involved in muscle repair, neurotransmitters, and anti-inflammatory mechanisms, and improve intestinal health, particularly during stress [64,76,77]. Collectively, these functions underscore the nutritional importance of NEAAs in maintaining intestinal integrity, immune resilience, and overall productivity in poultry nutrition.

### 2.3. Limiting Amino Acids and Their Roles in Poultry Growth and Health

Essential AAs play a central role in numerous physiological processes, including the synthesis of structural proteins, enzymes, hormones, and neurotransmitters [78]. An inadequate supply of one or more EAAs limits protein synthesis and consequently impairs growth performance. In plant-based poultry diets, methionine, lysine, threonine, tryptophan, and valine are generally considered the primary limiting amino acids [26]. Because AA requirements differ among poultry species and production stages, the recommended dietary levels of these key limiting AAs for broilers, layers, breeders, and turkeys are summarized in Table 2.

#### 2.3.1. Methionine

It is an essential sulfur-containing AA and is typically the first limiting AA in poultry diets [96]. In C-SBM-based diets, methionine falls below requirements to meet optimal performance, hence necessitating supplementation [97,98]. Methionine plays a critical role in protein synthesis, acting as a methyl group donor via S-adenosylmethionine and as a precursor for cysteine and carnitine synthesis [38,99]. According to Ak and Sözcü [100] and Bin et al. [37], methionine supports glutathione synthesis, an essential antioxidant that neutralizes ROS and protects immune cells during oxidative stress. Baker and Boebel [101] and Waldroup et al. [102] identified DL-methionine (DL-Met) and DL-2-hydroxy-4-methylthio butanoic acid (DL-OH-Met) as the two primary synthetic sources used in poultry diets to optimize methionine supply. Although both DL-Met and DL-OH-Met are used to correct methionine deficiency in poultry diets, their bioefficacy varies, depending on the form supplied and the mechanisms governing intestinal absorption.

The synthetic methionine is primarily supplied as powder DL-methionine (DLM; 99% purity) or liquid DL-methionine hydroxy analogue free acid (MHA-FA; 88% active substance), with the liquid form generally exhibiting 70–75% of the bioefficacy of the powder form on an equimolar basis [103,104]. The variation in utilization between DLM and MHA-FA arises from differences in their intestinal absorption mechanisms. L-methionine is absorbed through both energy-dependent and concentration-dependent pathways involving Na-dependent and Na-independent transport systems, whereas MHA-FA relies mainly on Na-independent but H-dependent transport systems [105,106]. This kinetic difference suggests that L-methionine has a higher affinity and maximal velocity for its transporter than MHA-FA, enabling quicker removal from the gut [107]. According to Rostagno and Barbosa [108], when broilers aged 21–42 days were reared under heat stress, the biological efficacy of liquid MHA-FA reached approximately 75% that of DL-methionine on an equimolar basis. In the same study, caecectomised cockerels exhibited higher net absorption for DL-methionine (97.2%) than for MHA-FA (90.8%), indicating reduced utilization efficiency of the liquid form under thermal stress. Similarly, Balnave and Oliva [109] observed that in broilers aged 3–6 weeks reared at constant (30 °C) or cyclic (25–35 °C) temperatures, DL-methionine showed improved feed conversion ratio (FCR) compared to liquid MHA-FA, while other performance traits were not different.

Alagawany et al. [110] reported that dietary methionine supplementation enhances productive performance, increases egg production, and improves egg quality in laying hens. In their 22-week trial with 160 Lohmann Brown layers (20–42 weeks of age), hens fed 0.72% total sulfur amino acid (TSAA) with 18% CP exhibited the highest egg production and increased feed efficiency, particularly between 26 and 30 weeks of age, compared with lower methionine levels. Similarly, Schutte and van Weerden [98] concluded that White Leghorn-type laying hens require approximately 390–440 mg methionine per day, within a TSAA intake of 775–800 mg/hen-day, to achieve peak egg production of 80–83 eggs/100 hen-day. Building on these layer findings, Swain and Johri [111] observed comparable body weight gains and feed efficiency in broiler chickens fed starter diets containing 0.37–0.87% methionine in a basal diet with 22.1% CP and 12.25 MJ ME/kg. In the same study, cellular immunity was significantly enhanced in birds receiving 0.65% methionine, while humoral immune responses were improved at 0.30% methionine when combined with higher choline supplementation. These immune benefits were evidenced by increased leucocyte migration inhibition values and higher antibody titres in ELISA tests.

There have been reports of methionine deficiency in poultry, leading to reduced feed intake, poor feed conversion, feather loss, and pecking [38,112]. In a 42-day broiler trial, Wu et al. [113] demonstrated that inadequate dietary methionine markedly reduced serum concentrations of IgG, IgA, and IgM, indicating a compromised humoral immune response. Conversely, excessive methionine supplementation can increase N excretion and cause environmental pollution [114]. These highlight the importance of precise dietary formulation.

#### 2.3.2. Lysine

It is widely recognized as the second limiting AA in C-SBM-based poultry diets [115]. Due to its pivotal role in protein metabolism, lysine is frequently used as the benchmark AA in the ideal protein concept to determine the relative requirements for other AAs [116]. This approach ensures a balanced AA profile that minimizes excesses, reduces catabolism of surplus AAs, and limits N excretion. Functionally, lysine is critical for protein synthesis, collagen formation, immune cell function, and overall growth performance in broilers [34,117]. Praharaj et al. [118] and Panda et al. [119] reported that starter diets containing only 1.10% lysine impaired antibody responses and humoral immunity in broilers. Panda et al. [119] further found that increasing the lysine level to 1.20% (20.77% CP) increased the feed intake and improved FCR. Additionally, maintaining a balanced AA profile with lysine at 1.30% (22.50% CP) during 0–21 days of age optimized broiler performance and immune response [118,119,120]. According to Kim et al. [80], broiler breeder hens aged 23–29 weeks fed diets containing 0.55% total lysine had reduced egg production, egg weight, and egg mass compared with those offered 0.71% lysine. Similarly, Kakhki et al. [121] reported that Hy-Line W-36 laying hens aged 32–44 weeks fed diets containing 0.657–0.857% digestible lysine showed improved egg production, egg weight, egg mass, and FCR, with optimal performance achieved at approximately 0.78–0.81% digestible lysine. Several studies have further demonstrated that both lysine deficiency and excessive supplementation can impair broiler performance, leading to reduced body weight, lower feed efficiency, diminished fat deposition, and reduced meat yield [122,123,124]. Consistent with these observations, Bastianelli et al. [125] demonstrated in a 10-day trial that broiler chicks fed a lysine-deficient diet (0.72% Lys) from hatch exhibited reduced feed intake, poorer feed conversion, and significantly lower breast muscle weight at both 3 and 10 days of age compared with those receiving adequate lysine levels (1.40% Lys). These findings confirm the detrimental effects of lysine deficiency on early growth and muscle development.

#### 2.3.3. Threonine

Threonine is an indispensable AA and is recognized as the third limiting AA in poultry diets [31]. Two forms of threonine exist: L-threonine, which is biologically active and utilized by poultry, and DL-threonine, a synthetic mixture containing both active L- and inactive D-isomers [126]. Threonine plays a key role in maintaining intestinal immune function and supporting optimal production performance in poultry. Azzam et al. [127] demonstrated that in Lohmann Brown laying hens (28–40 weeks of age) fed a low-CP diet (14.16% CP), supplementing L-threonine at 0.66% optimized egg production, egg mass, and FCR, with performance plateauing between 0.57% and 0.66%. In the same study, peak intestinal immune responses were observed at 0.66% and 0.74% digestible threonine, including increased ileal mucin2 (MUC2) and occludin mRNA expression as well as elevated secretory immunoglobulin (sIgA) mRNA levels, collectively indicating enhanced mucosal immunity. Faria et al. [128] demonstrated that Hy-Line W36 laying hens fed C-SMB diets containing 0.35–0.58% threonine showed improved egg production and egg mass, with optimal performance achieved at daily threonine intakes of approximately 439–462 mg/hen/day. Threonine also plays an essential role in protein synthesis, especially in the production of mucin, a glycoprotein that protects the intestinal lining and maintains gut integrity [129,130]. This role in mucin synthesis is reflected in structural improvements, as Zaefarian et al. [131] found that supplementing standardized ileal digestible threonine up to 0.7% in 0–21-day-old Ross 308 broilers significantly increased duodenal and jejunal weights, villus height, epithelial thickness, goblet cell numbers, and crypt depth across the duodenum, jejunum, and ileum. These parameters are directly linked to improved intestinal mucosal integrity and barrier function. However, this substantial contribution to mucin production means that threonine significantly influences endogenous AA secretions, thereby affecting the accuracy of overall AA digestibility [132,133]. Conversely, Wang et al. [134] reported that excess or deficiency of threonine can lead to reduced feed intake, slower growth rates, and compromised immune function. Similarly, Zhang et al. [46] reported that threonine deficiency in the culture medium upregulated the expression of IL-8, MUC2, and IgA; these effects were reversed upon threonine supplementation, highlighting its critical role in supporting a well-functioning local immune system in broiler chicks.

#### 2.3.4. Tryptophan

Tryptophan is recognized as an essential AA and the least abundant among the limiting AAs in laying hens [135,136]. Tryptophan is crucial for protein synthesis to sustain egg production [55]. In addition, tryptophan is a precursor for serotonin, a neurotransmitter that regulates mood, appetite, stress response, and behavior in poultry. n a review by Fouad et al. [7], the dietary tryptophan requirement for broiler chickens was reported to range from 0.16% to 0.20%, with stage-specific recommended level supporting feed intake, daily weight gain, and FCR of approximately 0.18% for starters [93], 0.198% for mid-growth [137], and 0.16% for finishing stage [138]. According to Dong et al. [139], 40-week-old Babcock Brown laying hens fed C-SBM diets supplemented with 0.2–0.4 g/kg L-tryptophan under summer conditions showed improved eggshell strength, serum albumin, serum immunoglobulin, and superoxide dismutase activity, indicating enhanced antioxidant status and immunity without affecting laying performance. Similarly, Son et al. [140] demonstrated that 70-week-old Hy-Line Brown hens fed C-SBM diets supplemented with 1.0% L-tryptophan showed improved egg production, egg mass, eggshell thickness, and feather condition, along with higher serotonin and lower corticosterone levels. Tryptophan was highlighted as a modulator of avian immunity through its metabolite melatonin, which promotes immune cell development [141]. Consequently, inadequate levels of tryptophan can lead to behavioral traits, such as reduced growth and feed efficiency, feather pecking and cannibalism. According to Mousavi et al. [135] and Wen et al. [142], feeding laying hen diets containing 1.56 g/kg or less tryptophan resulted in reduced feed intake.

## 3. Current Challenges in Optimizing Amino Acid Nutrition

Despite the recognized importance of AAs in poultry nutrition, several challenges persist in optimizing their dietary inclusion. One significant issue is the variability in AA requirements among different poultry species and production stages, which necessitates precise formulation of diets to meet specific needs [143,144,145]. Additionally, the bioavailability of AAs can be influenced by factors such as feed processing methods and the presence of anti-nutritional compounds, and these affect accurate assessment of dietary AA levels [146,147,148]. The environmental impact of N excretion resulting from protein metabolism is another challenge. Excessive nitrogen loss from N intake leads to ammonia emissions [149,150], which contribute to air and water pollution. Moss et al. [151] noted that optimizing AA profiles in diets can reduce N excretion, thereby mitigating environmental pollution. Beyond environmental concerns, economic factors related to AA supplementation and diet formulation also play a crucial role in poultry nutrition. Burley et al. [152] reported that the cost of SAAs, which are often used to adjust dietary AA deficiencies in poultry diets, can be a limiting factor, especially in resource-limited settings. Achieving both cost-effectiveness and nutritional adequacy remains a key challenge for poultry nutritionists.

Recent innovations in AA supplementation, particularly the development of chelated and encapsulated forms, are designed to improve bioavailability and metabolic efficiency [153]. For instance, Sun et al. [154] reported that broilers fed diets containing encapsulated L-lysine and DL-methionine at 60% of the crystalline AA levels used in the control diet exhibited increased villus height in the jejunum and maintained AA transporter expression without compromising performance, suggesting enhanced nutrient absorption capacity despite reduced supplementation. Similarly, Sun et al. [155] demonstrated that 47-week-old Hy-Line Brown hens fed diets supplemented with encapsulated L-lysine-HCl and DL-methionine at 80% CLM (control levels) maintained egg production and showed higher postprandial plasma lysine concentrations than controls, allowing a 20% reduction in SAA use without performance loss. Nonetheless, the effectiveness of these novel forms remains variable, which highlights the need for further research to determine optimal inclusion rates and delivery strategies.

The optimization of AA nutrition now faces emerging challenges driven by genetic, welfare, and regulatory shifts. Over recent decades, relentless selection for rapid growth and improved feed efficiency has pushed modern broilers and layers toward their physiological limits [156,157], leading to metabolic disorders that alter amino acid metabolism and tissue deposition. Rapid muscle accretion has been associated with myopathies such as woody breast, white striping, and spaghetti meat, which compromise carcass quality and consumers acceptance [158,159,160]. In broiler breeders, feed restriction, necessary to regulate reproductive performance, has become a welfare concern [161]. Carney et al. [162] reported that hens restricted to commercial body weight targets possess abdominal fat pads comprising only ~0.5% of body weight, indicating insufficient metabolic reserves to support sexual maturation.

In addition to biological and welfare constraints, regulatory changes such as the global ban on antibiotic growth promoters (AGPs) have reshaped nutritional strategies. Thomke and Elwinger [163] demonstrated that AGPs improved growth and feed efficiency by 3–5% in broilers, while Maria Cardinal et al. [164] observed that their withdrawal increased the FCR from 1.66 to 1.72, with accompanying economic and environmental costs. Therefore, the prohibition of AGPs has intensified the need for nutritional alternatives, including precision AA diets, to sustain gut health and performance. Collectively, these traditional and emerging challenges indicate that while AAs are indispensable for optimal poultry productivity, their dietary optimization requires a multifaceted approach, combining precise formulation, improved feed processing, novel delivery technologies, and continued research to refine AA metabolism and requirement models in modern production systems.

## 4. Feed Ingredients Supplying Amino Acids and Formulation Strategies

A comprehensive understanding of AA-rich feed ingredients, their composition, processing effects, and bioavailability is essential for formulating poultry diets that meet physiological demands and support optimal performance. Evaluating these ingredients based on their AA digestibility, anti-nutritional content, and interaction with digestive function allows for more precise and cost-effective protein nutrition.

To fulfill these AA requirements, poultry diets incorporate a variety of feedstuffs that differ in their essential and non-essential AA profiles; the summarized data are presented in Table 3 and Table 4. Commonly used feed ingredients such as soybean meal, canola meal, fish meal, peanut meal, and cottonseed meal provide varying AA contributions to support poultry growth and tissue development [165,166,167]. For instance, soybean meal, the most widely used plant-based protein source, supplies a substantial amount of lysine, threonine, tryptophan, and isoleucine, which are vital for muscle development and immune function [168,169]. In contrast, corn, although primarily an energy source, is deficient in lysine, threonine, and tryptophan, and also limited in methionine, necessitating strategic supplementation with protein-rich feed ingredients to balance its AA profile [170]. Fish meal serves as a valuable supplement for sulfur-containing AAs like methionine and cysteine, which are important for feather development and metabolic functions [171,172].

Strategic formulation is necessary to overcome the limitations of individual feed ingredients and ensure balanced AA inclusion. For instance, SBM provides an excellent source of lysine, its low methionine content often requires supplementation with fish meal or synthetic methionine to meet the poultry requirement [173,174,175]. Similarly, corn-based diets are often deficient in lysine and tryptophan, which can compromise protein synthesis and growth. Supplementing corn-based diets with lysine and tryptophan has been shown to enhance egg production, reduce feather pecking and stress-related behavior in laying hens [97,176]. Additionally, plant-based meals such as peanut and cottonseed can supply important AAs but often contain anti-nutritional factors such as gossypol or tannins that reduce bioavailability and require processing to improve nutrient utilization.

**Table 3:** Essential amino acid composition, limiting amino acids, and digestibility coefficients of common poultry feed ingredients.

Feed Ingredient	Major Essential AAs Present	Major AA Concentration g/kg (As-Fed Basis)	First Limiting Essential AAs (Relative to Requirement)	Digestibility	Reference
Corn	Leucine	10.8	Lysine	High for Leucine	[177,178]
Wheat	Leucine	8.4	Lysine	High for Leucine	[177,179]
Soybean Meal	Arginine	36.1	Methionine	High overall	[177,180]
Canola meal	Methionine, Leucine	25.2	Lysine	Varied	[179,181]
DDGS ^1^	Leucine	30.9	Lysine	High for Leucine	[182]
Fish meal	Lysine, Methionine	46.3	Threonine	High Lysine	[183]
Meat and Bone meal	Arginine	35.9	Methionine	Lower than blood meal	[177]
Blood meal	Lysine	118.9	Isoleucine	High for Lysine and Histidine	[177]
Feather meal	Leucine	70.3	Lysine	High in Histidine	[177]

Major essential amino acids refer to those present in relatively high concentrations within the listed feed ingredient. First limiting amino acids are those present in insufficient quantities relative to the amino acid requirements of poultry, based on requirement-to-supply ratios. Classifications are derived from ingredient profiles reported by Ravindran et al. [177]. ^1^ DDGS: Distillers dried grains with solubles; AA: amino acids.

**Table 4:** Non-essential amino acid composition and comparative variability among poultry feed ingredients.

Feed Ingredient	Predominant Non-Essential AAs	Major AA Concentration g/kg (As-Fed Basis)	Notable Variable	Reference
Corn	Glutamic Acid and alanine	16.3	Higher alanine compared to wheat	[177,179]
Wheat	Glutamic acid and Aspartic acid	32.8	Higher glutamate compared to corn	[177,179]
Soybean meal	Aspartic acid	87.3	Significant variations across Brazilian states	[179,180]
Canola meal	Aspartic acid and glutamic acid	62.7	Glutamic acid has higher apparent ileal AA digestibility	[177,181]
Fish Meal	Glutamic acid and aspartic acid	75.6	Higher glutamic acid compared to canola meal	[177,179,183]
Cottonseed meal	Glutamic acid	80.6	Higher proline compared to corn	[177,179]
Meat and bone meal	Glycine	68.7	Higher glycine and proline	[177,179]
Blood meal	Aspartic acid and leucine	100.1	Very high level of leucine compared to other animal sources	[177]
Sunflower	Glutamic acid	62.4	Lower glutamic acid compared to soybean meal	[177]
Feather meal	Glutamic acid and glycine	95.5	Very high glutamic acid	[177]

Predominant non-essential amino acids (NEAAs) refer to those occurring in the highest concentrations within each feed ingredient, based on total content expressed in g/kg DM. Notable variations refer to differences in NEAA concentrations compared to other common feedstuffs, as observed in data reported by Goldflus et al. [180], Huang et al. [179], and Ravindran et al. [177]. Values are representative of ingredient averages and may vary by geographic origin, processing method, and sampling technique. AA: amino acids.

### 4.1. Anti-Nutritional Factor in Feed Ingredients

Anti-nutritional factors (ANFs) are compounds present in feed ingredients that interfere with nutrient intake, availability, and utilization. In poultry diets, ANFs may affect growth performance and intestinal health by forming complexes with nutrients or digestive enzymes, thus reducing the digestibility of carbohydrates, proteins, and minerals [184,185,186,187]. The tolerance to ANFs varies among animal species. Different feedstuffs contain varying types and levels of ANFs (Table 5). For instance, SMB contain trypsin inhibitors and lectins [188,189], rapeseed or canola meal contains glucosinolates and tannins [190,191,192], cottonseed meal contains gossypol [193,194], and barley or wheat contain β-glucans and arabinoxylans [195,196,197], respectively.

#### 4.1.1. Trypsin Inhibitors

Trypsin inhibitors (TIs) are proteinaceous chemical compounds mostly found in SBM and other legume-based feedstuffs. These small proteins form stable complexes with trypsin, a key pancreatic protease, thereby blocking its enzymatic activity and impairing protein digestion [216]. This inhibition prevents the enzyme from hydrolyzing dietary proteins, resulting in reduced AA absorption and increased endogenous AA losses, particularly in young birds with immature digestive systems [217]. The presence of TIs in the diet triggers a compensatory response in the pancreas, leading to hypersecretion of digestive enzymes and eventual pancreatic hypertrophy [218,219]. According to Kumar et al. [21] dietary inclusion of purified TIs in raw soybean diets stimulated excessive pancreatic enzyme synthesis and caused notable hypertrophy in both rats and chickens.

Trypsin inhibitors in soybeans are primarily composed of two distinct families, Kunitz trypsin inhibitors (KTI) and Bowman-Birk trypsin inhibitors (BBTI), which account for approximately 80% and 20% of total TI content, respectively [220]. Kunitz TIs selectively binds and inhibits trypsin, while BBTI has dual specificity, inhibiting both trypsin and chymotrypsin simultaneously [221]. These inhibitors are considered the primary contributors to reduced protein digestibility in poultry diets [217]. Feeding trials have demonstrated that elevated levels of dietary TIs depress growth rate and feed intake in poultry, confirming their negative impact on performance [222].

Thermal processing remains the most effective strategy for mitigating the negative effects of TIs. Heat treatment denatures the tertiary structure of these inhibitors, rendering them inactive while preserving the nutritional quality of the feed. For instance, Krupa [223] demonstrated that controlled heating effectively reduces TI activity without compromising protein integrity. Similarly, Ram et al. [224], reported that boiling SBM at 100 °C for 15 min reduced TI activity by up to 95% as determined by laboratory assay of trypsin inhibitory activity, highlighting the importance of optimized processing conditions.

#### 4.1.2. β-Glucans

β-Glucans are soluble non-starch polysaccharides (NSPs) composed primarily of linear chains of β-(1,3) and β-(1,4)-linked glucose units [225]. They are particularly abundant in cereal grains such as barley and oats, with hull-less barley noted for its high extract viscosity due to elevated β-glucan content [226] and in yeast, algae, and fungi [227]. Andersson et al. [228] found that β-glucan content in barley ranges from 13% to 17% and 2.2% to 7.8% in oat. The level of β-glucan in cereal grains is affected by both genotype and environment [229]. Several studies have demonstrated that β-glucans increase digesta viscosity by forming gel-like networks in the small intestine, which limit enzyme-substrate interactions, slow gastric emptying, reduce nutrient diffusion, and increase microbial substrate availability [230,231]. Slama et al. [232] noted that high digesta viscosity slows the diffusion of endogenous enzymes, reducing their access to feed substrates and consequently limiting nutrient digestion. β-glucans are fermented by hindgut microbiota to produce short-chain fatty acids (SCFAs) such as acetate, propionate, and butyrate. These SCFAs reduce intestinal pH, stimulate beneficial bacteria such as *Lactobacillus*, *Bifidobacterium*, and *Enterococcus* species, enhance intestinal barrier integrity, and suppress the proliferation of pathogenic species [233,234,235,236]. To corroborate this, Tian et al. [237] reported that yeast-derived β-glucan supplementation in broiler chicks challenged with *Eimeria* and *Clostridium perfringens* improved gut morphology and integrity, reduced *C. perfringens* populations, and elevated antibody titers against both pathogens. These findings suggest that β-glucans enhance mucosal immunity and intestinal barrier function during enteric infections.

Beyond their fermentative role, yeast-derived β-glucans further act as immunomodulators, attenuating intestinal inflammation by downregulating Toll-like receptor (TLR)- mediated signaling and restoring immune balance, particularly in older or pathogen-challenged chickens [238,239]. This was supported by Cox et al. [240] who demonstrated that yeast-derived β-glucan supplementation in broiler chicks from hatch to 14 days of age modulated immune responses without affecting body weight gain or immune organ development. The dietary inclusion of β-glucan altered the cytokine-chemokine profile, particularly the ratios of interleukin (IL)-8, interferon-γ, IL-13, and IL-4, suggesting enhanced immune modulation under non-challenged conditions. When challenged with *Eimeria* species, β-glucan supplementation further elevated helper T-cell activity and proinflammatory cytokines, indicating improved innate and adaptive immune function. β-glucans slow intestinal transit, which often results in wet, sticky droppings that degrade litter quality and increase ammonia production [186], and increase the incidence of footpad dermatitis [241]. However, the use of exogenous enzymes has been shown to effectively attenuate the effect of β-glucans in poultry [242,243]. Amerah et al. [244] demonstrated that broilers fed a wheat-based diet supplemented with xylanase (2000 U/kg) for 42 days showed a 16% improvement in weight gain, a 6% reduction in FCR, and a 20% decrease in *Salmonella enterica* serovar cecal colonization. Similarly, Lázaro et al. [245] demonstrated that laying hens fed diets supplemented with a combination of xylanase, β-glucanase, and hemicellulase showed a 4.4% improvement in fat digestibility and a 2.5% increase in apparent metabolizable energy corrected for nitrogen (AMEn) due to reduced intestinal chyme viscosity, confirming the effectiveness of enzyme blends in alleviating β-glucan-induced antinutritive effects.

#### 4.1.3. Glucosinolates

Glucosinolates are nitrogen- and sulfur-containing thioglucosides that function as secondary metabolites in *Brassicaceae* plants and are considered anti-nutritional when present in animal feed [246]. These compounds are stored in plant cell vacuoles. Glucosinolates themselves are non-toxic; however, their anti-nutritional effects arise after enzymatic hydrolysis by β-thioglucosidase (myrosinase), producing reactive metabolites such as isothiocyanates, thiocyanates, nitriles, and oxazolidine-thiones [247,248]. Rapeseed (canola) meal and mustard cake are major dietary sources in poultry, with rapeseed meal ranking as the second-most used plant protein source after SBM [249,250]. The hydrolysis products confer a bitter flavor, which can reduce feed intake, and at high dietary levels have been linked with liver impairment [251,252], thyroid enlargement, and reduced plasma thyroid hormones [253]. Their main detrimental effect is goitrogenicity, resulting in the disturbance of thyroid hormone synthesis and secretion. Mechanistically thiocyanate anions directly compete with iodine for the sodium-iodine symporter (SIS) protein, inhibiting iodine binding to thyroglobulin, while oxazolidinethiones (e.g., goitrin) inhibit dimerization of diiodotyrosine (T2) into thyroxine (T4) and prevent triiodothyronine T3 and T4 release into the bloodstream [254]. Furthermore, goitrin flavin-containing monooxygenase-3 (FMO3), causes trimethylamine (TMA) accumulation in egg yolk and producing a “fishy taint”, especially in layers carrying a single-nucleotide polymorphism (SNP) in the FMO3 gene [255].

In layer hens, inclusion of rapeseed above 200 g/kg in the diet has been reported to increase liver hemorrhage and reduce egg production [256,257]. However, broiler chickens have exhibited variable responses to different dietary inclusion levels of canola meal [258]. Leeson et al. [259] reported that diets containing up to 38% canola meal in broilers and 25% in layers had no significant effect on feed intake, weight gain, or feed efficiency. Meanwhile, recent studies have reported that canola meal inclusion negatively affected weight gain, feed intake, and FCR [260,261]. This is likely due to variations in canola cultivar, processing conditions, bird genotype and age, as well as diet form and formulation. Modern “double-zero” canola cultivars have reduced glucosinolate content to <30 μmol/g in the defatted meal, thereby reducing liver toxicity [262,263]. Likewise, thermal processing to inactivate myrosinase, such as cooking at 60 °C, has been shown to reduce enzymatic hydrolysis [264]. Zhang et al. [265] demonstrated that fermentation of rapeseed meal with *Lactobacillus delbrueckii* and *Bacillus subtilis* (2:1 ratio) under controlled conditions (36 °C, 15% inoculum, 16% bran content, and a 1:1.5 feed-to-water ratio) effectively degraded glucosinolates by up to 94.6%, providing a practical biological detoxification strategy. Similarly, Ahmed et al. [266] reported that extrusion processing of canola meal significantly increased AMEn and improved ileal digestibility of crude protein and key AAs (Asp, Glu, Ser, Thr, and Trp), offering an effective means to enhance nutrient utilization and mitigate glucosinolate-induced anti-nutritional effects.

## 5. Advances in Amino Acid Utilization: Processing, Metabolism, and Microbiota Interactions

The bioavailability of AAs and the overall digestibility in poultry diets are greatly affected by the nutritional quality of feed ingredients. Different processing methods, such as cooking, autoclaving, germination, irradiation, spray- and freeze-drying, fermentation, and extrusion have been demonstrated to enhance plant protein quality by reducing ANFs and increasing AA release [267,268]. Fermentation has been shown to effectively reduce ANFs such as phytates and tannins, thereby enhancing the nutritional quality of oilseed meals, including soybean and cottonseed [269,270,271]. Similarly, the use of extrusion processing method has been found to be effective in enhancing the protein quality of soybean by effectively inactivating heat-labile anti-nutritional factors, including urease, trypsin inhibitors, and lipase, thereby improving overall nutritive value [272]. However, improper application of heat-based processes can compromise AA bioavailability. Excessive heating may initiate Maillard reactions, reducing lysine availability and diminishing the nutritional value of ingredients like SBM and field peas [273,274]. Plant-based feedstuffs naturally contain ANFs, including phytates, tannins, protease inhibitors, and saponins, which bind AAs to form complexes, and limit their absorption [275,276,277]. Enzyme supplementation has been widely adopted to counter these effects, particularly with phytase, protease, and carbohydrase. These enzymes enhance AA digestibility by breaking down protein-bound complexes and releasing nutrients otherwise inaccessible to the bird [278,279,280]. As emphasized by Ravindran [242], enzyme inclusion not only improves AA bioavailability but also reduces N excretion, contributing to more sustainable poultry nutrition. This aspect is discussed in detail in subsequent sections.

Beyond conventional processing methods and enzyme supplementation, emerging nutritional strategies such as in-ovo feeding have been developed to optimize AA utilization [281]. This approach delivers essential nutrients, including AAs, directly to the developing embryo prior to hatching [282], thereby enhancing early nutrient availability and supporting post-hatch growth and metabolic efficiency [283,284]. According to Ajayi et al. [285], in-ovo supplementation of cysteine, lysine, or their combinations in thermally challenged broiler embryos improved key physiological and performance outcomes. Specifically, administering 3.5 mg cysteine per egg enhanced hatchability, while a low-dose mixture of cysteine (1.7 mg/egg) and lysine (1 mg/egg) improved post-hatch growth performance, antioxidant status, and duodenal villus height. These targeted AA interventions during late incubation (day 18) not only support embryonic development under heat stress but also enhance immune function and growth efficiency in the post-hatch phase. In a related study, Eisa et al. [286] reported that in-ovo injection of L-threonine on day, 18 of incubation, followed by post-hatch dietary supplementation (3 g/kg), improved feed intake and FCR, increased relative spleen and thymus weights, and enhanced intestinal villus height-to-crypt depth ratio in Ross 308 broilers. In addition to AA delivery, in-ovo supplementation can also provide carbohydrates, which are naturally limited in eggs as well as probiotics and vaccines. These additions help address embryonic nutrient gaps, strengthen early immune resilience, and improve readiness for post-hatch growth [287,288,289]. Furthermore, this approach helps counter the developmental challenges posed by delayed post-hatch feed access [290].

Building on early-life interventions, a clearer understanding of AA metabolism is essential to support physiological regulation throughout the bird’s lifecycle. Amino acids support key physiological processes, such as protein synthesis, energy production, and neurotransmitter synthesis [291,292]. Among these, BCAAs, particularly leucine and isoleucine, contribute to protein deposition and regulate energy homeostasis, nutrient metabolism, and gut integrity. Their catabolism during periods of stress or nutrient deficiency provides an alternative energy source, demonstrating their metabolic flexibility in maintaining homeostasis [293,294]. According to Koopmans et al. [295] and Ruan et al. [296], tryptophan plays a dual role by supporting protein biosynthesis and serving as a precursor to serotonin and other bioactive metabolites that regulate behavior and stress response in poultry.

Amino acids play a pivotal role in shaping gut microbial composition and metabolic activity in poultry, adding complexity to their functional role beyond host-mediated metabolism. Intestinal microbes contribute to AA turnover by fermenting undigested proteins, synthesizing certain essential AAs, and modulating host metabolic pathways [34,297]. Undigested AAs entering the hindgut influence microbial fermentation patterns, short-chain fatty acid production, and pathogen proliferation [298,299]. Microbial proteases hydrolyze dietary proteins into peptides and free AAs, which can be incorporated into microbial protein or converted into bioactive compounds such as biogenic amines [300,301,302]. Specific AAs exert targeted effects on intestinal microbiota composition. Tryptophan modulates mucosal immunity via indole derivatives [303]. Threonine supports mucin synthesis, maintaining epithelial integrity and fostering beneficial bacteria [129]. Methionine enhances antioxidant status and increases *Lactobacillus* abundance [304,305]. Dietary lysine balance influences fermentation dynamics and butyrate production [306]. Understanding the interactions between hosts and their microbiomes is crucial for developing more targeted AA-based nutritional strategies that promote gut health and enhance production efficiency.

## 6. Exogenous Enzymes in Amino Acid Nutrition

In modern poultry production, protein sources are a major cost component in poultry feed, and improving AA digestibility is essential for optimizing feed efficiency and performance [307]. Because poultry lacks endogenous enzymes capable of degrading NSP and phytate, dietary supplementation with exogenous enzymes has become a key strategy to enhance nutrient utilization. These enzymes (Table 6), primarily produced from microbial sources such as *Escherichia coli*, *Aspergillus*, and *Bacillus* spp., are recognized as safe by the Codex Alimentarius and FAO/WHO standards [308]. The main classes include phytases, carbohydrases (fiber-degrading enzymes like xylanase, β-glucanase, β-mannanase, cellulase), and proteases, which improve AA availability by hydrolyzing complex carbohydrates, phytate, and protein-bound nutrients [309]. Their inclusion enhances nutrient absorption, reduces anti-nutritional effects, and increases overall feed efficiency [242,310].

### 6.1. Phytase and Amino Acid Digestibility

Phytase is one of the most widely utilized exogenous enzymes in poultry nutrition. It is supplemented in the feed to hydrolyze the phytate into myo-inositol and inorganic phosphorus (P) and free up the AAs that were previously bound [317]. Selle et al. [313] reported that phytase supplementation significantly improves protein and AA digestibility by reducing the formation of insoluble phytate-protein complexes, thereby enhancing their accessibility to digestive enzymes. According to Velázquez-De Lucio et al. [308], the enzymatic degradation of phytate not only liberates bound P but also enhances the digestibility and bioavailability of AAs and minerals, including calcium, zinc, and magnesium. Furthermore, phytate not only forms insoluble complexes with phosphorus and other cations but can also inhibit endogenous protease activity, contributing to increased AA losses. Cowieson and Ravindran [318] demonstrated that elevated levels of dietary phytic acid increase the total endogenous AA flow by more than 25%, and that this effect is almost entirely attenuated by inclusion of dietary phytase. In a related study, Cowieson et al. [319] documented that when male broiler chickens were fed diets containing elevated phytic acid levels (from 8.5 to 14.5 g/kg), there was a 68% increase in endogenous AA loss, highlighting its potent anti-nutritional effect. However, phytase has been shown to counteract these effects by enhancing protein solubility, reducing mucin secretion, and improving AA absorption [279,320]. Supporting this, Namkung and Leeson [311] demonstrated that when broiler chicks were supplemented with 1149 FTU/kg of phytase, ileal digestibility of total AAs and N digestibility improved by approximately 2%. Additionally, Cowieson et al. [321] reported that phytase supplementation at 150–300 FTU/kg significantly enhanced AA digestibility in broiler chicks, with valine, threonine, isoleucine, alanine, and aspartic acid showing notable improvements ranging from 4.5% to 5.6%. These results indicate that low doses of phytase are sufficient to improve the digestibility of specific AAs, particularly those susceptible to phytate-induced endogenous losses. However, it is important to note that the efficacy of phytase is influenced by both the dosage and type of diet. Ravindran et al. [312] observed that optimal improvements in AA digestibility were achieved at 500 FTU/kg in diets with medium to high phytate content for broiler chicks.

### 6.2. Protease Supplementation and Amino Acid Digestibility

Proteases catalyze the cleavage of peptide bonds, effectively converting complex proteins into smaller peptides and free AAs, and subsequently release trapped nutrients within protein-bound structures [322,323]. Common plant-derived feed ingredients such as SBM and sunflower meal are known to contain a variety of ANFs, including protease inhibitors like trypsin and chymotrypsin, saponins, and tannins, which bind to dietary proteins and AAs, and reduce their availability for digestion and absorption [188]. In a 21-day study with male broilers fed a C-SBM diet, da Nobrega et al. [310] demonstrated that supplementation with microbial protease at 30,000 NFP/kg significantly increased CP digestibility by 2.50% and improved the digestibility of essential and non-essential AAs by 2.64% and 2.52%, respectively. Additionally, the same study found that protease supplementation mitigated the negative effects of formaldehyde, reducing AA digestibility loss from 4.49% to 1.81%. It also exhibited synergistic effects with copper sulfate, further enhancing CP digestibility by 4.63% and AA digestibility by up to 5.28%.

Angel et al. [324] observed that supplementation of broiler diets with 75,000 PROT units/kg of monocomponent protease increased apparent CP digestibility by 6.1%, along with threonine (7.8%), arginine (3.5%), lysine (5.4%), and histidine (3.3%). In Arbor Acres broilers, Lee et al. [325] reported that feeding ProAct 360 protease at 50 g/MT improved total tract digestibility of methionine, phenylalanine, and valine. In a similar study, Liu et al. [326] demonstrated that supplementation with two commercial proteases, RONOZYME^®^ ProAct (15,000 PROT/kg) and ProAct 360™ (30,000 NFP/kg), significantly improved the SIAAD in 21-day-old male broilers fed SBM, with the improvements particularly evident for valine and isoleucine in birds receiving ProAct 360™. Poudel et al. [315] showed that supplementing 60 g/MT of protease in diets formulated at 90–95% dAA/CP significantly improved ileal digestibility of lysine, threonine, tryptophan, and valine in Hy-Line W-36 laying hens aged 30 to 50 weeks, thereby enhancing nutrient utilization under protein-restricted conditions. Furthermore, protease supplementation has been shown to enhance performance metrics, such as body weight gain and FCR in broilers [327], and to increase egg mass and overall profitability in laying hens [328].

### 6.3. Carbohydrase and NSP Degradation

Carbohydrase enzymes play a pivotal role in poultry nutrition by addressing the inherent inability of non-ruminant animals to fully digest complex carbohydrates present in plant-based feed ingredients [308,329]. There are two major fiber-degrading enzymes: xylanase and β-glucanase. Ojha et al. [309] noted that xylanase hydrolyze arabinoxylans found in wheat and rye, while β-glucanase degrades β-glucans present in barley and oats, respectively. Other notable carbohydrases include amylases, which specifically target soluble sugars like starch [330], and these enzymes function by degrading complex carbohydrates into simpler sugars, effectively breaking down plant cell wall components and thereby facilitating access to encapsulated nutrients [314,331]. Stefanello et al. [332] demonstrated that xylanase supplementation improves both N and AA digestibility by lowering digesta viscosity in broiler chicks. Several other studies also reported a positive association between reduced digesta viscosity and improved N digestibility. For instance, improved N utilization was noted in ducks and turkeys [333,334,335], while enhanced AA digestibility was documented in broilers [336,337]. In a review by Moita and Kim [338], they demonstrated that adding xylanase enzyme to broilers’ diet reduced the jejunal viscosity by an average of 37%, with an 8% average daily gain. Sun et al. [339] reported that broilers fed diets supplemented with 1800 BGU/kg of β-glucanase showed improved body weight gain and FCR, likely due to the reduction in digesta viscosity facilitated by the enzyme inclusion. Shalash et al. [340] reported that supplementing corn-distillers dried grains with soluble diets with Kemzyme plus dry^®^ at 250 g/ton improved ether extract digestibility, egg production percentage, egg number, and egg mass in 30-week-old Inshas laying hens. A similar study by Ribeiro et al. [341] found that dietary supplementation of barley-based diets with 1000 U/kg recombinant microbial β-1,3-1,4-glucanase from day 1 to 28 increased feed intake and improved growth performance in broiler chickens, unlike a 1,4-β-glucanase, which was ineffective in vivo. While xylanase supplementation improves digestibility, its efficacy varies depending on diet type. Kiarie et al. [342] reported that xylanase is more effective in wheat-based diets due to their higher xylan content, resulting in greater improvement in digesta viscosity compared to corn- or barley-based diets. These findings highlight carbohydrase vital role in optimizing nutrient utilization across avian species and feed types.

### 6.4. Synergistic Effect of Enzyme Combinations

Exogenous enzymes can exhibit synergistic effects, where the combined impact of multiple enzymes is greater than the sum of their individual effects. This phenomenon is crucial for maximizing the nutritional value of feed ingredients in animal production, as different enzymes can target distinct ANFs or facilitate each other’s activity. The synergistic effects of exogenous enzymes have been well-documented in poultry nutrition, particularly when xylanase and phytase are co-supplemented in wheat-based broiler diets. Ravindran et al. [316] reported that this combination increased AME by 19.0% and enhanced ileal digestibility of 14 AAs by 8.6%, exceeding the improvements from either enzyme alone. In the same study, AAs such as lysine, histidine, and glycine demonstrated synergistic gains beyond additive expectations. Likewise, Selle et al. [313] confirmed these findings in broilers from 1 to 21 days post-hatch, reporting that the combined supplementation of xylanase and phytase improved ileal digestibility of 17 AAs, with notable synergistic responses for arginine, alanine, cystine, and tyrosine. The combination also enhanced N digestibility, restored growth performance in low-phosphorus diets, and improved sodium absorption. This effect was attributed to xylanase increasing substrate permeability and reducing gut viscosity, thereby facilitating phytase action and improving nutrient availability.

Beyond carbohydrase-phytase interactions, protease combinations have also demonstrated synergy. According to da Nobrega et al. [310] co-supplementing protease with copper sulfate in broilers enhanced CP digestibility by 4.63% and AA digestibility by up to 5.28%, a response not achieved with copper alone. Similarly, protease inclusion mitigated the adverse effects of formaldehyde, reducing AA digestibility losses from −4.49% to −1.81%. In laying hens, Poudel et al. [315] observed that diets supplemented with both protease and phytase improved the ileal digestibility of lysine, threonine, valine, and tryptophan, especially under reduced protein conditions, suggesting phytase’s role in liberating protein-phytate complexes and enhancing protease efficacy. To corroborate this, Bauer et al. [343], reported that supplementation of broiler diets with increasing levels (0, 100, 200, and 300 g/ton) of a multi-enzyme blend (Allzyme^®^ Spectrum) led to a quadratic increase in ileal digestibility of arginine, histidine, and valine compared to the negative control. Specifically, average ileal digestibility of total essential amino acids improved by 2.63% and non-essential amino acids by 2.07% at the optimal 200 g/ton inclusion level, relative to the negative control treatment. 

However, factors such as enzyme type and diet composition influence the consistency of synergistic responses. Olukosi et al. [330] noted that while phytase improved performance in poultry fed marginal C-SBM diets, adding a xylanase-amylase-protease (XAP) enzyme blend containing xylanase (2500 U/kg), amylase (400 U/kg), and protease (4000 U/kg) produced no additional benefit unless paired with phytase. In contrast, Woyengo et al. [344] reported that supplementing C-SBM-based diets for broiler chickens with combinations of phytase (1500 FTU/kg), amylase (80 KNU/kg, and a cocktail of non-starch polysaccharide-degrading enzymes (NSPase) (75 g/metric ton) produced synergistic effects on the ileal digestibility of both energy and protein. Nevertheless, not all enzyme combinations yield synergistic outcomes. Amerah et al. [244] reported that inclusion of XAP blend in C-SBM-based diets in broiler improved growth performance and dietary energy utilization beyond what was observed with individual enzymes; however, the effects were only partially additive and not synergistic. These findings suggest that successful enzyme synergy depends on a precise combination of enzyme types to dietary substrate, particularly under nutrient-reduced or high-fiber diets.

In addition, the efficacy of exogenous enzymes in AA nutrition is significantly influenced by both bird age and genetics [345,346]. The effects of feed enzymes on intestinal functionality necessarily vary with age due to the rapid development and subsequent changes in the digestive system that occur post-hatch [347]. Consequently, the neonatal chick, whose rapid growth may be limited by intestinal development, is often considered the most responsive to enzyme application [348,349]. Olukosi et al. [350] demonstrated that in both broilers and turkeys, xylanase supplementation improved FCR at 28 days, while the effect of phytase on phosphorus digestibility was more pronounced at 7 days than at 28 days, indicating that younger birds exhibit a greater enzymatic response owing to higher intestinal development. Similarly, Ravindran et al. [351] reported that xylanase supplementation in wheat-based diets improved growth performance across three broiler strains (A, B, and C), with Strain A exhibiting the highest ileal AA digestibility coefficient (0.858) and Strain B the lowest (0.791), confirming genotype-dependent variation in enzyme efficacy.

### 6.5. Mechanisms of Action: How Exogenous Enzymes Improve Amino Acid Digestibility

Exogenous enzymes enhance poultry’s AA digestibility through several mechanisms. Primarily, these enzymes function by directly hydrolyzing complex ANFs and otherwise indigestible feed components. Phytases dephosphorylate phytate, liberating bound AAs and phosphorus [329]. Carbohydrases like xylanases and β-glucanases brea down NSPs, which bind nutrients and increase digesta viscosity [309,352,353], and proteases directly hydrolyze dietary proteins and various protein-bound ANFs, making AAs more readily available [310,324,354]. Consequently, this breakdown compromises the integrity of the plant-based feed cell wall and lowers digesta viscosity, thereby enhancing nutrient release and absorption [355]. A reduction in digesta viscosity enhances nutrient digestibility by facilitating closer interactions between endogenous enzymes (e.g., trypsin, chymotrypsin) and their substrates, thereby improving the efficiency of hydrolysis along the small intestine [342,356]. This reduced viscosity also accelerates the digesta passage rate while optimizing retention time in the upper gastrointestinal tract, ensuring that nutrients are thoroughly exposed to enzymatic action before reaching the hindgut. As a result, less undigested material reaches the hindgut, reducing the growth of pathogenic bacteria such as *Salmonella* and coliforms [357], lowering total anaerobic counts [358], and promoting beneficial *Lactobacillus* populations [359,360], ultimately enhancing gut health. Furthermore, enzymes play a crucial role in reducing metabolic endogenous nutrient losses. Cowieson and Bedford [361] reported that phytase supplementation mitigates the adverse effect of phytate on endogenous AA flows and sodium secretion. Proteases, in particular, also contribute to overall digestibility, and some evidence suggests they can support gut morphology [325,362], although specific studies on small intestine morphometry may show no significant effect [325,328]. Additionally, the synergistic and additive effects of combining carbohydrases with phytase enhance phytate degradation, thereby improving amino acid and nutrient utilization while reducing nutrient waste [313,316].

### 6.6. Data-Driven Insights—Role of Exogenous Enzymes in Amino Acid Digestibility and Nutrient Utilization in Poultry

Research has consistently highlighted the benefits of exogenous enzyme supplementation in poultry nutrition (Table 4). For example, protease supplementation has been shown to increase AA digestibility, leading to significant improvements in growth performance and feed efficiency [363]. According to Zavelinski et al. [364], protease supplementation improved FCR and increased ileal digestible energy (IDE) regardless of corn batch, although the apparent ileal digestibility (AID) of dry matter and CP remained unchanged. In a related study, Vieira et al. [365] reported that protease supplementation significantly enhanced the ileal digestibility of DM and CP compared to diets without protease, with IDE further increasing upon inclusion of 30,000 protease units. Their findings further revealed that the novel serine protease effectively compensated for reduced digestible lysine (6%) and metabolizable (20 kcal/kg AME) in broiler diets, a compensatory effect that was more pronounced in diets formulated for moderate growth, particularly those containing animal protein. Although Lin Law et al. [366] observed minimal impacts on growth performance, carcass traits, and physiological responses in broilers under heat stress when protease was added to low-CP and/or ME diets, they suggested that microbial protease supplementation could still serve as a valuable nutritional strategy to lower dietary ME or CP levels, which potentially reduce abdominal fat accumulation, improve feed efficiency, and profitability [367,368].

Similarly, the supplementation of exogenous carbohydrases has been associated with improved performance in broiler chickens. Cowieson et al. [369] reported a significant improvement in FCR and body weight gain in broilers fed corn-SBM diets supplemented with xylanase and glucanase without affecting feed intake. This enhancement was accompanied by a notable increase in IDE at 42 days, indicating improved feed efficiency. In addition, Jeichitra and Srinivasan [370] found that xylanase supplementation enhanced the digestibility of hemicellulose and energy in corn-based diets, while Purushothaman et al. [371] demonstrated that mannanase supplementation in a corn-soy diet maintained comparable growth under low-energy conditions, thereby reducing feed cost per kilogram of weight gain. Building on these findings, Govil et al. [372] investigated multicarbohydrase supplementation (xylanase at 50 mg/kg + mannanase at 50 mg/kg + amylase at 40 mg/kg) in broilers. They observed improvements in overall performance and CP digestibility, particularly in birds fed diets marginally deficient in energy. Together, these studies underscore the role of carbohydrases in enhancing nutrient utilization, growth performance, and feed efficiency. Beyond carbohydrases, phytases play crucial roles in improving AA digestibility and protein utilization. Ravindran et al. [373] demonstrated that supplementing broiler diets with fungal phytase at 500 FTU/kg increased the average AID coefficients of 15 AAs by 4.34% (0.775 vs. 0.809), with a higher inclusion of 1000 FTU/kg further increasing digestibility by 5.68% (0.775 vs. 0.819) in birds fed a wheat-SBM-based diet at 28 days post-hatch. In a follow-up study, Ravindran et al. [312] reported that broiler starters fed C-SBM-based diets supplemented with 1000 FTU/kg of *Escherichia coli*-derived phytase (Phyzyme XP) showed significant improvements in AID of AAs, including 8.3 percentage units increase for cystine and 2.5 for arginine, with an average increase of 4.0 units across all AAs, suggesting enhanced protein utilization and potential reductions in N excretion. A similar study by Amerah et al. [374] examined bacterial phytase at 1000 FTU/kg in C-SBM diets at 21 days post-hatch and reported a 12.30% increase (0.748 vs. 0.840) in the ileal digestibility coefficients of 17 AAs, with EAAs such as methionine and threonine improving by 5.45% (0.880 vs. 0.928) and 15.7% (0.661 vs. 0.765), respectively. A more recent study by Martínez-Vallespín et al. [375] also evaluated bacterial phytase at 1000 FTU/kg, showing a 10.30% increase (0.738 vs. 0.814) in the average ileal digestibility of 17 AAs at 21 days post-hatch in diets comprising corn, SBM, and sunflower meal. Additionally, Dersjant-Li et al. [376] applied modeling techniques to predict AA digestibility responses and estimated that 2000 FTU/kg phytase would increase the mean AID coefficients of 18 AAs by 6.58% (0.76 vs. 0.81). A systematic review conducted by Cowieson et al. [377], which synthesized data from 24 studies spanning 1996–2006 on broiler chickens, confirmed that phytase supplementation increased AA digestibility coefficients from around 2% to 4%. Collectively, these insights underscore the critical role of exogenous enzyme supplementation in optimizing AA digestibility and nutrient utilization in poultry production.

## 7. Ileal Amino Acid Digestibility Measures

Historically, protein quality and AA availability in animal feed were measured using excreta digestibility values. However, as Ravindran et al. [378] noted, this approach had several limitations in poultry due to extensive caeca fermentation, which alters protein digestion, and contamination from urinary N. Consequently, ileal AA digestibility was widely accepted as a more accurate and sensitive method, as it measures AA absorption at the terminal ileal level prior to microbial modification in the hindgut [379]. This methodological shift represents a critical advancement in nutrient estimation, providing a more direct assessment of nutrient utilization. Within the ileal digestibility framework, three distinct measures are recognized: AID, SIAAD, and true ileal digestibility (TID) [19]. While AID includes both undigested dietary and endogenous AAs secreted into the gastrointestinal tract, it often underestimates digestibility, especially in low-protein diets. Moreover, AID values lack additivity in mixed feed formulations, making them less suitable for precise diet optimization [380,381]. In contrast, SIAAD corrects AID values for basal endogenous losses (BEL) to improve accuracy and additivity across ingredients, while TID accounts for both basal and diet-specific losses, providing the most precise estimate of AA utilization [382,383]

### 7.1. Standardized Ileal Amino Acid Digestibility

Standardized ileal amino acid digestibility is a measure of AA bioavailability and is calculated by subtracting the basal ileal endogenous AA losses from the total ileal AA outflow [19]. This measure has emerged as a more practical and reliable approach to address the lack of additivity drawbacks with the digestibility of AA in feed formulation. In a review by Adedokun et al. [384], they emphasized that SIAAD is achieved by correcting AID values for basal endogenous AA losses. The term “standardized” means only the BEL are subtracted from the ileal outflow. Jansman et al. [385] categorize endogenous losses into basal or specific. Basal endogenous losses are influenced by total dry matter intake but independent of the type of diet [386]. Conversely, specific endogenous losses are induced by dietary factors such as fiber and anti-nutritional compounds [19]. Trypsin inhibitors in SBM stimulate pancreatic secretions, while insoluble fiber enhances mucin production, and soluble viscous fiber increases microbial protein flow to the ileum [217]. Several studies reported that SIAAD values are likely additive in mixed diets for swine and poultry [387,388,389]. Supporting this, Cowieson et al. [363] observed that the use of AID values led to approximately 7% underestimation of AA digestibility in C-SBM diets, while the prediction error decreased to around 1% when SIAAD values were applied. The additivity of SIAAD values makes it more accurate for ileal AA digestibility estimate than TID and AID. Also, it allows for the precise incorporation of various ingredients, even those with lower digestibility, into cost-effective and nutritionally balanced diets.

### 7.2. Basal Ileal Endogenous Amino Acid Losses

Basal endogenous losses are defined as the unavoidable AA losses that are not influenced by the specific composition of the feed ingredient being consumed [390,391]. These losses originate from the animal’s normal physiological processes and gut tissue turnover, including secretions such as digestive enzymes, bile acids, unabsorbed mucus, and sloughed intestinal cells [19,392]. They constitute the minimum AA losses that an animal experiences regardless of dietary composition. Basal endogenous losses are primarily influenced by the total dry matter intake (DMI) of the animal, which is why they are often expressed in relation to DMI [386,393]. The most widely used method for determining these losses involves feeding a nitrogen-free diet (NFD) [394,395], although it is acknowledged that this method may place the animal in an unnatural physiological state. Other methods are highly digestible protein (HDP) diets [396], or the regression method [397,398]. However, results can vary due to factors like age, species, and collection methods. According to Adedokun et al. [399] and Adedokun et al. [400], basal ileal endogenous AA expressed per kilogram of DMI declines with age in both broiler chickens and turkeys. In the same studies, the values recorded on day 5 post-hatch were notably higher than those observed on day 15, after which they remained relatively constant on day 21. The NFD is still widely considered the most consistent and reliable approach for correcting apparent AA digestibility [401,402]. The determination of BEL is fundamental for standardizing AID values, as this correction overcomes AID’s non-additivity and yields more accurate, additive digestibility coefficients crucial for precise feed formulation.

### 7.3. Specific or Diet-Induced Ileal Endogenous Amino Acid Losses

Specific endogenous losses are additional AA losses that are directly induced by particular characteristics or components within a feed ingredient or diet, occurring above the basal level [19,380]. These losses are primarily caused by the presence of certain dietary factors, such as high fiber content and various ANFs, including phytate, TIs, lectins, tannins, and glucosinolates. For instance, Kluth and Rodehutscord [403] observed that high dietary cellulose in broiler diets increased endogenous AA losses, although the AA composition remained unchanged. Phytic acid, a common ANF, has been shown to increase mucin and overall endogenous AA losses in broiler chickens [318]. These diet-induced secretions contribute to a reduction in the apparent digestibility of dietary AAs. Supporting this, Ravindran et al. [378] demonstrated that reduced apparent AA digestibility observed in wheat-barley-based diets may be attributed to xylan or beta-glucan-induced stimulation of ileal endogenous amino acid (IEAA) efflux. Despite their significant impact on nutrient utilization and metabolic cost, reliable and routine procedures for directly measuring these specific endogenous losses are not yet widely available, making their practical quantification challenging [390]. This limitation is the primary reason why TID, which would account for all such losses, is currently impractical for routine feed formulation. 

### 7.4. Apparent Ileal Amino Acid Digestibility

Apparent Ileal Digestibility is a measure of AA digestibility, calculated by subtracting the total ileal outflow of AAs from the total dietary AA intake. This value represents the net disappearance of AAs from the digestive tract proximal to the distal ileum [19]. A significant characteristic of AID is that the total ileal outflow includes both undigested dietary amino acids and endogenous AAs (encompassing both basal and specific losses) that have been secreted into the gastrointestinal tract and not reabsorbed. A major limitation of AID is its inability to distinguish between undigested dietary and endogenous AAs. This often results in an underestimation of AAs digestibility coefficients, especially in diets with low protein content or poorly digestible proteins, where basal endogenous losses contribute more substantially [384,404]. Consequently, AID values are not consistently additive in mixed feed formulations, making them less reliable for precise diet optimization compared to standardized systems [382,387]. This inconsistency primarily stems from the non-linear response of apparent digestibility to changes in dietary AA concentration, such as when varying the proportion of a test ingredient in an NFD [405].

### 7.5. True Ileal Amino Acid Digestibility

True Ileal Digestibility (TID) conceptually represents the most fundamental measure of AA digestibility, aiming to quantify only the AAs genuinely absorbed from a feed ingredient. This is achieved by correcting AID values for all endogenous AA losses, which are classified into both basal (diet-independent) losses, associated with metabolic functions and mucosa turnover, and specific (diet-induced), which arise in response to dietary components such as fiber or anti-nutritional factors, e.g., phytate, protease inhibitors [390,392,394]. Although TID theoretically provides an intrinsic and constant value for each feed ingredient, facilitating direct comparisons across various dietary conditions [380], its practical use is limited. This limitation arises from the absence of routine and reliable methods to measure specific endogenous losses, which are highly variable and diet-dependent [390]. Consequently, TID values often lack additivity in mixed feed formulations, reducing their applicability in precision diet formulation [19]. According to Yoon and Kong [406], in a study conducted with Ross 308 male broiler chickens, the measured AID values for 5 out of 17 AAs in a C-SBM mixed diet were significantly different from their predicted values, indicating a lack of additivity. In a review by Adedokun et al. [384], he noted that the term “true digestibility” has occasionally been used interchangeably with SIAAD, despite important differences between the two. However, recent consensus clearly distinguishes SIAAD, which corrects AID for basal endogenous losses only, from TID, which additionally accounts for diet-specific losses [380,394,404,406]. Given that basal endogenous losses can be consistently estimated, mostly through NFD, SIAAD provides more additive digestibility coefficient values than TID, thereby reinforcing its adoption as the preferred metric for estimating AAs quality [363,407].

## 8. Factors Affecting SIAAD in Poultry

Several factors influence SIAAD (Table 7) and, consequently, the accuracy of endogenous AA digestibility estimates in poultry diets. These factors must be carefully considered when formulating diets for optimal performance.

### 8.1. Feed Processing

Feed processing methods can significantly influence AA digestibility by altering the physical and chemical characteristics of feed ingredients. Common techniques such as pelleting, extrusion, grinding, dehulling, and thermal treatment have been employed to improve feed efficiency, though their effects are highly dependent on processing intensity and ingredient type [421,422]. For instance, grinding enhances digestibility by increasing the surface area available for enzymatic hydrolysis [408,409], whereas pelleting has been shown to reduce AA digestibility due to heat-induced changes such as protein denaturation or the formation of indigestible complexes with NSP [423,424].

Thermal processing plays a dual role. On one hand, it is necessary for the inactivation of heat-labile ANFs, such as trypsin inhibitors found in SBM and other legume-based feedstuffs [425]. This is supported by findings from de Coca-Sinova et al. [426], who reported that soybean products with elevated trypsin inhibitor levels exhibited reduced apparent digestibility of cystine. This reduction was attributed to increased endogenous secretion of cystine, likely due to its abundance in trypsin molecules that become bound and secreted in response to inhibitor activity. On the other hand, excessive heat application may lead to protein damage through mechanisms such as Maillard reactions, AA racemization, and protein cross-linking, particularly in the presence of reducing sugars [427,428]. Lysine, in particular, is vulnerable due to its reactive ε-amino group [429]. Adedokun et al. [414] reported that excessive heating during processing significantly reduced lysine’s AID, especially in dark distillers’ dried grains with solubles (DDGS). Similarly, studies have shown that overheating or autoclaving SBM can result in the formation of D-isomers, such as D-lysine and D-methionine, which are poorly absorbed and reduce both true digestibility and biological availability in poultry and swine [274,413,425,428]. The lysine content of cottonseed meals is particularly prone to degradation under heat, due to its reactive ε-amino group [430], while variability in meat meal digestibility has been linked to differences in raw material composition, rendering duration, and temperature [431].

Pelleting is a hydrothermal process that involves compressing feed through die openings using heat, steam, moisture, and pressure to form compact pellets [432]. This process improves AA digestibility by denaturing protein structures and inactivating heat-sensitive ANFs, thereby improving enzymatic access and nutrient absorption [433]. However, thermal intensity must be carefully controlled. Excessive heat during pelleting can damage the proteins and reduce their bioavailability [273]. Boltz et al. [411] reported that conditioning C-SBM that contains DDGS diets at 77 °C, 82 °C, and 88 °C for 30 or 60 s improved AA digestibility at moderate conditions (82 °C for 30 s). However, excessive thermal exposure (88 °C for 60 s) led to a decline in digestible AA concentrations. These findings were corroborated by Samarasinghe et al. [434], who found that increasing the conditioning temperature from 60 °C to 75 °C improved feed intake by 6% and body weight gain by 9%, but further heating to 90 °C led to reductions in both parameters. In the same study, N and metabolizable energy utilization declined by 4.0% and 3.2%, respectively.

Extrusion is another feed processing method that utilizes high temperature, moisture, pressure, and mechanical shear to transform the feed structure [435]. This process gelatinizes starch, denatures proteins, and disrupts ANFs, thereby enhancing digestibility [412]. Supporting this, Ahmed et al. [266] evaluated extruded canola meal in broilers and found a significant improvement in apparent metabolizable energy (9.39 vs. 10.87 MJ/kg) and in the ileal digestibility AAs such as glutamic acid, threonine, serine, and tryptophan. Stein and Bohlke [436] also reported improved SIAAD of all AAs in extruded field peas. Likewise, Clarke and Wiseman [437] demonstrated that extrusion enhanced lysine digestibility in full-fat soybeans, though the extent of improvement depended on processing temperature. In their study, male Ross broiler chicks (day 19–26) fed diets containing extruded full-fat soybeans processed at increasing barrel temperatures of 90–160 °C exhibited an increase in ileal apparent digestible lysine from 10.53 to 17.63 g/kg, alongside an improvement in the coefficient of ileal apparent digestibility (CIAD) from 0.58 to 0.86. Clearly, when properly optimized, pelleting and extrusion enhance AA availability, improve N retention, and reduce fecal N excretion to support environmentally sustainable poultry production.

Other processing strategies, including dehulling and soaking, have also demonstrated nutritional benefits. In a study by Schulze et al. [438], dehulling improved nutrient utilization by removing fiber-rich seed coats containing ANFs such as tannins, although excessive dehulling could compromise nutrient stability. Schöne et al. [439] demonstrated that grinding and soaking, followed by drying, enhanced the feeding value of rapeseed for growing pigs by promoting the breakdown and leaching of glucosinolates, a key ANF in rapeseed. In addition, Bedford et al. [440] observed that pelleting, when supplemented with enzyme, altered intestinal viscosity and improved broiler performance on rye-based diets, though the exact impact on SIAAD or IEAA was not clearly outlined. These findings emphasize that feed processing can improve AA availability and reduce endogenous losses by enhancing protein solubility and minimizing anti-nutritional effects when appropriately optimized. However, excessive thermal exposure, particularly during rendering or drying, can significantly impair digestibility, especially for heat-sensitive AAs like lysine. Therefore, ingredient-specific and condition-dependent processing protocols are essential to maintain nutritional value and accurately apply SIAAD values in precision feed formulation.

### 8.2. Type of Feed Ingredient

The composition of feed ingredients plays a central role in modulating endogenous AA losses at the terminal ileum, particularly due to their impact on mucin secretion, enzyme output, and gut epithelial turnover. A study by Ravindran and Hendriks [441] revealed that broilers fed nitrogen-free or enzyme-hydrolyzed casein diets consistently exhibited high ileal flows of glutamic acid, aspartic acid, threonine, and glycine. These AAs were confirmed to be dominant constituents of endogenous secretions, primarily from mucin glycoproteins and bile, which are relatively resistant to proteolysis. Similarly, Kluth and Rodehutscord [403] observed that in broilers fed diets varying in CP and cellulose levels, inevitable losses of glutamic acid, aspartic acid, and threonine remained the most pronounced, irrespective of dietary fiber inclusion, indicating a conserved endogenous protein profile. In a study by Kong and Adeola [415], broilers offered NFD with varying dextrose-to-corn starch ratios showed the highest ileal losses for glutamic acid, followed by aspartic acid and threonine, which together accounted for more than 18% of total AA flow. This pattern reinforces their physiological dominance in endogenous output. Whitehouse et al. [442] reported that glutamic acid, aspartic acid, threonine, leucine, serine, valine, and proline made up approximately 70% of total ileal endogenous protein in broilers, largely due to their abundance in mucins and digestive enzymes. 

Importantly, while fiber-rich ingredients such as cellulose were shown to increase the quantity of endogenous AA losses, the relative composition of those losses remained stable [403]. A similar report was also echoed by Parsons et al. [443], who noted a greater AA and glucosamine excretion in roosters fed high-fiber and NFDs. In addition, studies by Adedokun et al. [416] and Kong and Adeola [415] demonstrated that NFDs formulated with dextrose and high dietary electrolyte balance induced greater endogenous AA flows, particularly glutamic acid, tryptophan, tyrosine threonine, and proline, than corn starch-based diets, likely due to enhanced mucosal stimulation and mucin release. In broilers, Adedokun et al. [414] demonstrated that increasing dietary calcium up to 3% in a NFD elevated total ileal endogenous AA losses by 39%, with tryptophan (74%), tyrosine (66%), glutamic acid (53%), and histidine (53%) showing the most significant increases, highlighting the role of high dietary calcium in stimulating mucosal secretions and mucin-associated amino acid excretion. These findings clearly suggest that the repeated identification of glutamic acid, tyrosine, threonine, and related amino AAs across diverse studies, regardless of diet type, strongly supports their origin from within the gastrointestinal tract, particularly from mucins and digestive enzymes. At the same time, dietary components such as fiber, and protein concentration can influence the total amount of endogenous protein lost from the gut’s metabolic processes.

### 8.3. Age

Ileal endogenous amino acid flow and digestibility coefficients are strongly influenced by the birds’ age. Adedokun et al. [396] and Adedokun et al. [399] reported that total AA and IEAA levels in 5-day-old broiler chicks and turkey poults were significantly higher than those measured at day 15 or 21. Specifically, the total AA level decreased by 53% in broiler chicks and 68% in turkey poults between days 5 and 15. Although Adedokun et al. [396] initially suggested that poults exhibited up to threefold higher IEAA flow than chicks when fed NFD or HDP diets, later data clarified that poults had approximately 1.35 to 2.2 times higher flow, depending on the diet [444]. In a related study, Adedokun et al. [445] further emphasized that age-related differences have been interpreted as a key reason for lower AID values observed in starter-phase birds, due to substantial endogenous losses. Barua et al. [379] further supported these findings by showing that IEAA level peaked at day 7, declined sharply on day 14, remained stable until day 35, and then dropped again on day 42. These patterns were attributed to decreased mucin secretion, prolonged digesta retention, and improved reabsorption of secreted AAs.

While the impact of age appears to decline as birds mature, Adedokun et al. [446] reported no significant differences in IEAA flow between 21-day-old broilers and 37-week-old layers when fed NFD or HDP diets. However, in the same study, They observed that 104-week-old caecectomised roosters recorded IEAA flow rates that were 3.5–12-fold higher than in both broilers and layers, suggesting a resurgence of endogenous output in aged birds. Ravindran and Hendriks [441], using enzyme-hydrolyzed casein (EHC), found that age-associated increases in endogenous losses could also persist for several AAs in broilers. Huang et al. [417] demonstrated that AID coefficients of AAs in different feed ingredients, including maize, sorghum, soybean meal, and meat and bone meal, increased progressively with age in 14-day, 28-day, and 42-day-old male broiler chickens. Furthermore, applying digestibility values from adult birds to younger ones should be done cautiously, as endogenous AA flows may differ [441].

### 8.4. Gut Microbiota

Gut microbiota and overall gut health significantly impact SIAAD. The intestinal microbiota, which primarily consists of Firmicutes and Bacteroidetes, plays an essential role in protein breakdown and AA absorption by fermenting non-digestible compounds and producing SCFAs [299,447]. Disruptions in gut microbiota due to diet, medications such as antibiotics, or environmental stressors (e.g., stocking density, heat or cold stress, litter quality, etc.) can lead to dysbiosis characterized by a loss of beneficial bacteria, increased pathogen colonization, and metabolic imbalances that ultimately affect nutrient absorption [448,449]. Coccidiosis, caused by *Eimeria species* (a protozoan), is a major gut health challenge in broilers, leading to over $10.4 billion losses annually [450]. Dahiya et al. [451] recorded that coccidiosis reduced feed intake, impaired growth, and increased susceptibility to necrotic enteritis. Similarly, Amerah and Ravindran [419] demonstrated that administering a mixed *Eimeria* spp. to a challenged male Ross 308 broiler chickens at day 14 resulted in elevated intestinal lesion scores and significantly reduced the AID of all measured AAs at day 21. The observed reduction in AA digestibility was directly associated with increased lesion severity, suggesting that compromised gut integrity contributes to diminished nutrient absorption. Adedokun et al. [452] and Kim et al. [418] also reported that coccidiosis significantly affects SIAAD by reducing digestibility and altering AA metabolism in broiler chicks. In a related study, Persia et al. [453] observed that acute *Eimeria acervulina* infection significantly reduced the AID of AAs, especially Threonine, Valine, Isoleucine, Lysine, and Arginine in chicks fed a C-SBM diet, with the most pronounced effects occurring within the day 12 to 13 post-infection. Given the significant influence of these factors on SIAAD, maintaining optimal processing conditions, selecting appropriate feed ingredients, and managing birds’ health and gut microbiota are essential for enhancing AA digestibility and overall poultry performance.

## 9. Environmental Impact of Amino Acid Nutrition in Poultry Production

Amino acid nutrition plays a pivotal role in poultry production, influencing not only the growth and health of the birds but also the environmental footprint of the industry [1]. As a result, there is an increasing focus on formulating low-protein diets and precision nutrition by incorporating low-cost crystalline AAs or alternative animal protein sources [454]. The relationship between AA intake and N excretion is central to understanding and mitigating the environmental impacts associated with poultry farming [1]. Nitrogen, a component of AAs, is metabolized in poultry and excreted mainly as nitrogenous waste products like urea and uric acid [455]. The efficient utilization of AAs in poultry diets can minimize N excretion, thereby reducing its environmental impact [11,456]. Therefore, exploring the connection between AA nutrition and N excretion is important to reduce environmental impact through optimized AA nutrition.

Nitrogen is a fundamental component of AAs, essential for protein synthesis, and plays important roles in metabolic processes in poultry [457]. However, excessive N excretion is a major environmental concern, contributing to ammonia emissions, acidification, and water pollution [458,459]. According to Cappelaere et al. [11], supplementing reduced-CP diets with feed-grade AAs enhances N utilization, minimizes environmental N losses, and supports optimal growth performance. Such et al. [455] also added that feeding low-CP diets reduces N excretion and, consequently, manure N content. However, when the supply of AAs in the diet exceeds the bird’s metabolic requirements, the surplus N is converted into waste products, such as uric acid, which is excreted in the manure. Conversely, inadequate AA intake impairs protein synthesis, reducing growth rates and feed efficiency. A balanced diet ensures that birds receive the optimal amount of AAs for growth, egg and meat production, and overall health, without generating excess N that burdens the environment. Additionally, the efficiency of N metabolism is influenced by factors such as the AA composition of the diet, the digestibility of feed ingredients, and the physiological status of the birds [455,460]. Overall, optimizing AA nutrition is a key strategy for reducing N excretion and mitigating the environmental impact of poultry production.

### 9.1. Strategies to Reduce Environmental Impact Through Optimized Amino Acid Nutrition

Several strategies can be employed to optimize AA nutrition in poultry and, consequently, reduce the environmental impact of N excretion.

#### 9.1.1. Precision Feeding and Diet Formulation

This method involves formulating diets that closely match the AA requirements of poultry at different stages of life by accurately determining the AA needs of poultry and ensuring that these requirements are optimally met. Pope et al. [461] showed that switching broiler diets every 24 h at the finisher stage (42 to 63 days) supported maximum growth performance while limiting oversupply of digestible AAs. In their study, broilers fed a sequence of 11 phase-fed diets with progressively decreasing levels of lysine, SAA, and threonine achieved comparable weight gain, feed efficiency, and carcass yields to birds fed a single NRC-based diet, despite consuming lower levels of digestible AAs. The reduction in AA oversupply, particularly digestible lysine and SAA, without compromising growth, demonstrates the potential of phase-feeding regimens to optimize nutrient use and minimize N excretion under practical feeding conditions. Corroborating their findings, Pope et al. [462] reported that a phase-feeding strategy in broiler diets from 43 to 63 days of age significantly reduced CP intake and N excretion, without compromising carcass composition or growth performance. Additionally, recent studies have demonstrated that low-CP diets supplemented with EAAs can effectively reduce N excretion while maintaining optimal growth performance [458,463]. Precision feeding not only improves N utilization but also enhances feed efficiency and reduces the environmental footprint of poultry production [151].

#### 9.1.2. Use of Synthetic Amino Acids

Synthetic amino acids such as DL-methionine, L-lysine, and L-threonine are routinely used to improve the AA profile of low-protein poultry diets [365]. Their inclusion reduces the need for high-protein feedstuffs, thereby lowering N excretion [464]. Therefore, supplementing reduced-CP diets with SAAs has been shown to maintain growth while minimizing environmental impact. In a studies conducted by Aletor et al. [465] and Bregendahl et al. [466], they reported that for each 1% reduction in dietary CP, N output was reduced by 10%. In a similar study, Belloir et al. [467], using optimized digestible threonine-to-lysine and arginine-to-lysine ratios, observed a 13% drop in N excretion per 1% CP reduction. These findings were corroborated by a meta-analysis from de Rauglaudre et al. [468], who reported a 10.4% decline in daily N excretion per 1% CP reduction, with no loss in N retention. Attia et al. [469] further demonstrated that reducing CP from 18% to 15% in finisher diets, while supplementing with methionine and lysine, did not impair carcass yield or meat quality but reduced N excretion by 21%. According to Woyengo et al. [470], supplementing low-CP broiler diets (190 and 170 g/kg CP in the starter and finisher phases, respectively) with crystalline AA, lysine, methionine, threonine, and valine reduced N excretion by approximately 12 g per bird from the starter to the finisher phase, while a further reduction to 170 and 150 g/kg CP combined with additional glycine-equivalent supplementation lowered excretion by about 16 g per bird. These results affirm that SAA supplementation enables precise protein formulation, maintaining growth while minimizing N waste.

#### 9.1.3. Alternative Protein Sources

The use of alternative protein sources, such as insect meal [471,472], black soldier fly larvae (*Hermetia illucens*) [473], mealworms (*Tenebrio molitor*) [474], grasshopper (*Acrida cinerea*) [475] and cricket larvae meal (*Acheta domesticus*) [476], offers a sustainable approach to improving AA profiles in poultry diets while reducing the environmental footprint associated with SBM. These insect-based proteins generally provide highly digestible essential AAs in sufficient quantities, thereby improving N utilization and minimizing excretion losses [472]. Wang et al. [475] reported that the AA profile of grasshoppers (65.4% CP), including methionine (1.70%), cysteine (0.69%), and lysine (3.79%) on a DM basis, closely matches that of fish meal, underscoring their potential as a nutritionally comparable alternative protein source for poultry diets. Similarly, black soldier fly larvae surpassed SBM in digestibility for most AAs, highlighting their potential as a viable and sustainable feed ingredient [473]. In addition to insect-derived proteins, several plant-origin alternatives, such as microalgae, have also gained attention as sustainable protein sources in poultry nutrition. Abdel-Wareth et al. [477] stated that microalgae, such as *Spirulina* or *Chlorella*, are rich in protein (50–70% CP) with a favorable AA profile, compared to SBM. Interestingly, they can be cultivated on non-arable land, utilizing waste nutrients and capturing carbon dioxide, thereby improving sustainability. Additionally, these alternative protein sources can be locally produced, reducing the need for imported feed ingredients and further enhancing the sustainability of poultry production. However, due to the presence of chitin, which contains non-protein nitrogen, CP content of insect meals can be overestimated when the conventional conversion factor of 6.25 is applied [478]. Excessive inclusion levels may also impair nutrient digestibility and growth performance because chitin can limit protein and AA availability in the gastrointestinal tract [479]. Nonetheless, long-term evaluations are required to quantify the true environmental and nutritional trade-offs of insect and algae-derived proteins compared with SBM.

### 9.2. Case Studies on Environmental Benefits of Improved Amino Acid Nutrition

Studies in poultry nutrition have demonstrated that optimizing AA intake by reducing dietary CP levels can yield significant environmental benefits without compromising performance. In a study by Askri et al. [480], they found that lowering CP by up to 2.0%, achieved by partially replacing SBM and corn gluten meal with alternative protein sources like meat and bone meal (MBM), effectively reduced N intake and excretion while maintaining growth performance in broilers. Similarly, Belloir et al. [467] showed that optimizing the AA profile reduced CP levels in growing-finishing male broilers to 17% without negatively affecting growth or meat quality. Furthermore, Kriseldi et al. [481] demonstrated that supplementing reduced dietary CP levels with adequate EAAs, reduced N excretion in broiler chicks. In their first trial, broilers fed a low-CP diet containing 24.0% CP exhibited a 14.1% reduction in N excretion compared to those receiving a 25.8% CP diet at 15 to 16 days of age, and a marked decrease in plasma uric acid concentrations. In a subsequent trial, birds fed reduced CP diets (ranging from 21.8% to 20.2%) had significantly lower plasma uric acid levels at day 21 compared to those on a 23.9% CP diet. Notably, the study established that dietary CP could be safely reduced from 23.9% to 20.0% during the starter phase (1 to 14 days and from 23.9% to 21.7% across the 1 to 21-day period, without compromising growth performance, when the diets were supplemented with DL-methionine, L-lysine, L-threonine, L-valine, glycine, L-isoleucine, L-arginine, and L-tryptophan.

Similar environmental benefits have been observed in layer hens. Heo et al. [482] found that reducing dietary CP in pullets and layers linearly decreased N excretion without affecting growth, organ weights, and the age and time to reach 50% egg production. Summers [483] reported that a diet with 11% CP reduced N excretion by up to 40% compared to a standard 17% CP C-SBM diet, with minimal impact on egg mass output. Since high protein intake is often needed for maximum egg size, these findings suggest that optimizing rather than maximizing egg production is a more sustainable strategy. The use of these dietary approaches will support environmental sustainability and, at the same time, maintain optimal performance in poultry.

## 10. Methods for Determining Amino Acid Requirements

Determining the precise AA requirements for poultry is essential for formulating effective and efficient diets. Several methods have been developed to estimate these requirements, each offering unique insights based on research context and practical considerations.

### 10.1. Nitrogen Balance Studies

Measure the difference between N intake and excretion to assess protein retention and utilization, thereby indicating AA adequacy [484].

### 10.2. Integrated Growth and Energy Model

Estimates growth using models (e.g., Gompertz functions for body and feather protein), applies allometric scaling to combine components, calculates daily AA needs (for both maintenance and gain), and determines dietary concentrations based on predicted energy (feed) intake [485].

### 10.3. Indicator Amino Acid Oxidation (IAAO) Method

Tracks the oxidation of a labeled indicator AA, where a plateau in oxidation rates indicates that additional amounts do not enhance utilization, thus pinpointing the requirement [486].

### 10.4. Graded Supplementation Method

Involves incrementally adding the test AA to a basal diet deficient in that AA and measuring performance responses until additional supplementation yields no further benefits [487].

### 10.5. Summit Dilution Method

Involves gradually diluting a summit feed, one that contains an excess of all AAs except the test AA (the first limiting), using either a non-protein diluent or another feed that provides the remaining AAs in proportions matching those in the summit feed [488].

### 10.6. Slope-Ratio Assay

Compares the slopes of performance responses (e.g., growth rate) between a reference diet and test diets with varying AA levels to determine the relative potency and adequacy of the AA [401].

Collectively, these methods provide valuable information for determining AA requirements in poultry, thereby ensuring that diets meet the birds’ nutritional needs in a cost-effective manner.

## 11. Recent Technological Advancements in Amino Acid Nutrition

In recent years, technological advancements have revolutionized AA nutrition in poultry production, significantly enhancing feed efficiency and sustainability. Traditional methods, such as the N equilibrium approach, have demonstrated limitations in accurately estimating AA requirements; hence, advanced computational models have been adopted for more precise nutrient formulation [489,490]. One major breakthrough is the development of factorial and growth-modeling techniques, which estimate AA requirements by partitioning nutrient needs into maintenance and growth components [491,492]. These models integrate large datasets and predictive algorithms, thereby refining feeding strategies to ensure that modern broiler strains receive optimized nutrition.

Recent advances in precision nutrition have introduced dynamic modelling techniques to estimate AA requirements in poultry more accurately. These models integrate diverse biological and environmental variables, such as age, body weight, growth rate, and sex, to refine nutrient delivery and optimize feed efficiency. For example, Wang et al. [145] developed a factorial-based dynamic model, coupled with the comparative slaughter technique, to predict the standardized ileal digestible AA requirements of broilers throughout the growth cycle. Their approach accounted for maintenance needs, protein deposition, and sex-specific differences, offering a more responsive and individualized feeding strategy for modern broiler production. Pomar et al. [493] and Zuidhof [494] reported that growth-modeling technologies now facilitate the prediction of daily nutrient requirements, supporting adaptive feeding strategies based on real-time data. Furthermore, Lange and Huber [495] have also developed a dynamic AA requirement model for broilers. Despite these advancements, there is a need for continuous research in AI-based nutrition optimization to ensure cost-effective diet formulation that mitigate environmental impact.

Another technological breakthrough is the increased use of single-cell protein (SCP) as an alternative protein source [496]. They are derived from microorganisms such as yeast, bacteria, and fungi. Single cell-protein has become a valuable component in poultry diets due to its high AA content [497,498]. Research on SCP production has primarily focused on optimizing production processes, enhancing its nutritional profile, and reducing associated costs. Consequently, SCP has gained traction in poultry diets because of its sustainability, cost-effective protein source that is capable of meeting the increasing global demand for animal protein [499]. Despite its high nutritional value, SCP presents challenges such as high nucleic acid content and digestibility concerns [500].

Additionally, precision nutrition, an essential component of precision livestock farming, can further enhance AA nutrition. By integrating biotechnological, metabolomic, computational, and protein engineering approaches, producers can adjust nutrient intake with greater accuracy, leading to optimized feed use, improved poultry health and productivity, and reduced environmental impact. Additionally, Cambra-López et al. [501] propose two potential tools for identifying AA imbalances in broilers, both individually and in groups. First is the use of serum uric N content as a rapid biomarker for AA imbalances, and secondly, the design and modeling of de novo proteins that are fully digestible and tailored to the animal’s requirements. Collectively, these technological advancements in AA nutrition not only promise improved poultry performance but also support enhanced environmental sustainability through more efficient feed use and reduced N waste.

## 12. Future Research Directions

The future of AA nutrition research presents several promising directions. One key area of focus is the exploration of alternative protein sources, such as plant-based proteins and insect meals. As the demand for poultry feed continues to rise, the need for sustainable and cost-effective alternatives to traditional protein sources like SBM is becoming increasingly urgent [502]. In this context, it will be essential to investigate the nutrient composition and AA balance of these alternative protein sources, as well as assess their sustainability and commercial production potentials.

Another promising research avenue is the role of the gut microbiome in AA metabolism [503]. Studies indicate that gut microbiota plays a crucial role in nutrient absorption, including AAs [504]. Understanding how different microbial communities affect the digestion and absorption of AAs could lead to innovative strategies for enhancing poultry health and performance. For instance, probiotic supplementation and prebiotic interventions could be employed to modulate the microbiome in ways that improve AA absorption and overall feed efficiency. Another interesting research area is the adoption of AI-driven monitoring of poultry health and feed intake, which will allow producers to fine-tune AA supplementation in real time, optimizing nutrient efficiency and preventing over-supplementation. This technological advancement could be particularly important in mitigating the environmental impact of poultry farming by reducing N and P excretion, which are major contributors to environmental pollution [13]. Collectively, these research directions promise to drive significant breakthroughs in AA nutrition, ultimately leading to more efficient, sustainable, and environmentally friendly poultry production systems.

## 13. Conclusions

Optimizing AA nutrition is essential for improving poultry performance, nutrient utilization, and environmental sustainability. Multiple dietary and physiological factors, including fiber, protein sources, anti-nutritional compounds, endogenous losses, gut health, and feed processing directly influence SIAAD. Strategic interventions, such as exogenous enzyme supplementation, chelated or encapsulated AAs, and precision nutrition models, have shown significant promise in enhancing digestibility and reducing N excretion while maintaining optimal bird performance. As formulation and feeding technologies advancements continue to evolve, future research should focus on refining dose-response relationships, validating matrix values across feedstuffs, and translating these innovations to commercial production. Integrating science-driven strategies with precision management can help meet global demands for efficient, sustainable poultry systems.

## Figures and Tables

**Table 1:** Summary of the classification and key physiological functions of essential and non-essential amino acids in poultry.

Amino Acid	Classification	Functions in Poultry	Reference(s)
Methionine + Cysteine	Essential	Supports protein synthesis and feather development; serves as a methyl donor in DNA methylation; maintains antioxidant defense via glutathione and taurine pathways	[41,42]
Lysine	Essential	Required for muscle protein accretion and collagen formation; enhances immune response; promotes egg formation	[43,44]
Threonine	Essential	Serves as a major component of mucin; supports immune-protein synthesis and lipid metabolism; maintains gut barrier integrity	[45,46]
Tryptophan	Essential	Precursor of serotonin and melatonin; regulates feed intake and stress response; reduces feather pecking.	[47,48,49]
Arginine	Essential	Precursor of nitric oxide; required for cell proliferation and wound healing; enhances immune response and reduces carcass fat deposition.	[50,51]
Isoleucine	Essential	Involved in muscle metabolism and repair; supports hemoglobin formation and immune function.	[52,53]
Valine	Essential	Promotes muscle protein synthesis; essential for feather keratin and egg albumen formation.	[54,55]
Phenylalanine	Essential	Precursor for tyrosine; contributes to feather pigmentation and thyroid hormone synthesis	[56,57]
Histidine	Essential	Required for histamine synthesis; maintains muscle pH buffering; supports oxygen transport in erythrocytes	[58,59]
Alanine	Non-essential	Facilitates glucose-alanine cycle in muscle; contributes to nitrogen transport and gluconeogenesis.	[60]
Aspartate	Non-essential	Involved in nucleotide synthesis and transamination reactions; contributes to arginine biosynthesis and neurotransmission.	[61,62]
Glutamate	Non-essential	Key precursor for glutathione; serves as a major excitatory neurotransmitter; provides energy for intestinal enterocytes and nitrogen for other AAs.	[63,64,65]
Glycine	Non-essential	Required for collagen and heme synthesis; essential for feather keratin structure; exhibits anti-inflammatory properties.	[66,67]
Serine	Non-essential	Precursor for glycine and phospholipid synthesis; supports DNA and protein biosynthesis.	[1,68]
Proline	Non-essential	Supports collagen and feather keratin synthesis; provides energy for intestinal cells; contributes to eggshell membrane integrity and strength.	[66,69]

AA: amino acids; DNA: Deoxyribonucleic acid.

**Table 2:** Dietary limiting amino acids requirement of Poultry.

Limiting Amino Acids	Species	Age	Strain	Requirements (% of Diet) or mg/g/Birds	Reference (s)
Lysine	Broiler	0–3 weeks 3–5 weeks	Cobb 500	1.22 1.16	[79]
	Broiler breeder	23–29 weeks	Ross 308	0.7–0.77	[80]
	Turkey	72–83 days 84–95 days	Tom Poult (Male)	0.68–0.67 0.53–0.51	[81]
	Layer	32–48 weeks	Dekalb White	660–70 mg/g/b	[82]
L-Methionine + Cystine	Broiler	0–10 days 11–23 days 24–35 days	Ross 308 (Male broiler)	0.69 0.66 0.62	[83]
	Layer	22–36 weeks	N/S	0.31	[84]
Methionine	Broiler	7 days 14 days 21 days	Ross 308	0.62 0.55 0.50	[85,86]
Threonine	Broiler	22–42 days	Ross 308 (Male broiler)	0.74	[87]
	Broiler	1–18 day	Arbor Acre Classic	0.71–0.72	[88]
	Layer	29–39 weeks	White Leghorn	0.42–0.43	[89]
	Breeder	60 weeks	Cobb strain broiler breeder	569 mg/g/b	[90]
	Turkey	0–3 weeks 3–6 weeks 6–9 weeks	Large White Turkey	0.97 0.88 0.77	[91]
Valine	Layer	39–40 weeks 41–60 weeks	Hy-line W36	0.53–0.77 0.86–0.87	[92]
	Broiler	28 days	Ross 308	0.90	[85,86]
Tryptophan	Broiler	1–18 days 22–42 days	Ross 308 Cobb 500 (male)	0.16–0.17 0.19–0.22	[93] [94]
	Layer	60–70 weeks	Dekalb White Layer	0.19	[95]

Values represent approximate dietary amino acid requirements expressed either as a percentage of diet or milligrams per gram of bird (mg/g/b), derived from Council [86] and recent literature. Requirements correspond to optimal growth or production performance under standard feeding conditions. Actual values may differ in relation to bird strain, age, dietary energy density, or environmental temperature, which were not specifically compared in this summary. N/S: Not stated

**Table 5:** Anti-Nutrients in common feed ingredients and their effects on poultry performance.

Feedstuffs	Anti-Nutrients	Effect on Birds’ Performance/AA Utilization	References
Soybean Meal	Trypsin inhibitors	Inhibit proteases, reduce protein digestion and absorption of AA	[198]
	Lectins	Lectins damage the gut lining, impairing nutrient uptake	[199]
	Saponins	Reduce feed intake and affect protein digestibility	[200]
	Phytic acid	Phytate binds minerals and reduce AA digestibility	[201]
Cottonseed Meal	Gossypol	Gossypol decreases feed intake, reduces egg production and quality, damages organs, and binds lysine.	[193,202,203]
	Cyclopropenoid fatty acids (CPFA)	In laying hens, affect egg discoloration such as brown yolk and pink albumen	[194,204]
Canola/Rapeseed Meal	Glucosinolates	Glucosinolates cause bitter taste, lower feed intake, thyroid dysfunction (goiter)	[205]
	Tannins	Tannins bind with proteins and affect growth performance	[32,206]
	Phytate	Phytate reduces minerals & AA availability.	[201,207]
Field Peas	Trypsin inhibitors	Activate pancreatic hypertrophy and reduce protein absorption	[208,209]
	Oligosaccharides	Cause digestive disorders, including mild diarrhea, possibly reducing feed efficiency	[210]
Barley	β-glucans	Increase intestinal viscosity in chicks, sticky feces, decrease nutrient digestibility and feed conversion ratio.	[211,212]
Wheat	Arabinoxylans	Arabinoxylans in wheat cause high gut viscosity, reducing digestibility of protein and energy.	[186,213]
Faba Bean	Trypsin inhibitors & Lectins	Reduce feed intake, egg production, body weight and reduces total protein and AME in broilers and laying hens	[214,215]

AA: amino acid.

**Table 6:** Effects of exogenous enzymes on amino acid digestibility, nutrient utilization, and performance in poultry.

Enzyme Type	Species	Diet	Measured Outcomes	Mechanism of Action	Reference
Phytase	Broilers	Chick-starter diet	↑ Ileal AA digestibility at 1149 FTU/kg—(~2%) Specifically, ↑ Val, Ile. Also, ↑ N digestibility (numerical increase of 2%).	Hydrolyzes phytate-protein complexes, which otherwise limit AA utilization. Improves AA release and reduces endogenous N loss.	[311]
Phytase	Broilers	C-SBM	↑ Ileal AA digestibility (averaging 4.0% units) at 1000 FTU/kg, ranging from 2.5% to 8.3% units for arginine and cystine respectively.	Improves AA digestibility by phytate hydrolysis, overcoming the anti-nutritive effects of phytic acid. Effectiveness varies with dietary phytate concentration and dosage. Partial dephosphorylation may suffice for AA digestibility, with higher doses primarily aiding phosphorus retention	[312]
Carbohydrase (Xylanase)	Broilers	Wheat-SBM	↑ Ileal digestibility of 13 AAs, including Histidine, Threonine, leucine, Aspartic acid, Glutamic acid, Serine (from 2.5% to 13.4%). ↑ AID of N (average 4.8%). ↑ Feed efficiency (6.2%).	Disrupts non-starch polysaccharides; enhances enzyme-substrate contact and, consequently, AA digestibility. Counteracts increased gut viscosity induced by soluble arabinoxylans	[313]
Carbohydrase (β-Glucanase)	Broilers	Barley-based	↑ Body weight gain, ↓ Feed conversion ratio when β-glucanase (CtGlc16A) was supplemented in barley-based diets.	Reduces digesta viscosity by cleaving mixed-linked glucans. This improves nutrient digestibility and absorption, and increases feed passage rate.	[314]
Protease	Laying hens	Commercial laying hen diet	↑ Apparent ileal digestibility of Crude Protein, Lysine, Threonine, Tryptophan, and Valine. Protease inclusion also reduced feed consumption and FCR.	Hydrolyzes complex dietary proteins into smaller peptides and free AAs, facilitating absorption. Degrades proteinaceous ANFs and protein-bound ANFs. It can supplement endogenous peptidase activity and reduce protein turnover.	[315]
Xylanase + Phytase	Broilers	Wheat-SBM	↑ AID of 14 AAs (average 8.7%) (or 15–17 AAs by average 8.6%); ↑ Apparent Metabolizable Energy (19%). The combination also restored BWG and improved FCR	Xylanase enhances cell wall permeability, releasing encapsulated nutrients and reducing digesta viscosity, which facilitates phytase action and nutrient absorption. Phytase hydrolyzes phytate, which is an integral component of the cell wall matrix, thus disrupting it. This leads to a synergistic increase in AA digestibility.	[313,316]

AAs: amino acids; AID: apparent ileal digestibility, FCR: feed conversion ratio; ANF: anti-nutritional factors; N: nitrogen; BWG: body weight gain. ↑: increase, ↓: decrease.

**Table 7:** Summary of factors affecting SIAAD in poultry.

Factor Type	Specific Example(s)	Mechanism of Impact	Key Amino Acids Affected	Reference(s)
Feed Processing	Grinding	Enhances enzymatic hydrolysis via increased surface area	General improvement in AA digestibility	[408,409]
	Pelleting (moderate vs. excessive)	Moderate heat improves digestibility; excessive heat causes protein damage	Lysine, Cystine	[410,411]
	Extrusion	Denatures proteins, disrupts ANFs, gelatinizes starch	Lysine, Threonine, Serine, Tryptophan	[266,412]
	Autoclaving (soybean meal)	Forms D-isomers → reduced true digestibility	D-Lysine, D-Methionine	[413]
	Excessive rendering/drying	Damages heat-sensitive proteins	Lysine, others	[274]
Feed Ingredient	High dietary calcium	Stimulates mucosal secretions → ↑ mucin-associated AA excretion	Tryptophan, Tyrosine, Glutamic acid, Histidine	[414]
	High-fiber or cellulose diets	↑ mucin secretion and gut turnover → ↑ endogenous losses	Glutamic acid, Threonine	[403]
	Nitrogen-free or casein diets	Stimulate mucin and bile secretion → ↑ endogenous AA flow	Glutamic acid, Aspartic acid, Threonine, Glycine	[415,416]
Age	Broiler chicks vs. poults	Younger birds → higher mucin secretion and IEAA flow → ↓ AID	Glutamic acid, Threonine, Aspartic acid	[396,399]
	Broilers at 14, 28, 42 days	Digestibility improves with age	All measured AAs	[417]
Gut Microbiota	Coccidiosis (*Eimeria* spp.)	Intestinal lesions → ↓ AID of all measured AAs	Lysine, Threonine, Isoleucine, Arginine, Valine	[418,419]
	Antibiotic or heat stress	Dysbiosis → ↓ nutrient absorption	Broad AA impact (non-specific)	[420]

This table summarizes key factors influencing standardized ileal amino acid digestibility (SIAAD) in poultry. Mechanistic effects are described based on experimental observations and general physiological principles. AA: Amino acids; ANFs: Anti-nutritional factors; IEAA: Ileal endogenous amino acid; AID: Apparent Ileal digestibility. ↑: increase, ↓: decrease, →: lead to.

## Data Availability

No new data were created or analyzed in this study. Data sharing is not applicable to this article.

## Abbreviations

AA	Amino acid
AID	Apparent Ileal Digestibility
AGFs	Antibiotic growth promoters
ANFs	Anti-nutritional factors
BCAA	Branch-chain amino acids
BIEAAL	Basal Ileal endogenous amino acid losses
BBTI	Bowman-Birk trypsin inhibitor
C	Corn
CP	Crude protein
DDGS	Distillers dried grains with solubles
DNA	Deoxyribonucleic acid
DM	Dry matter
DMI	Dry matter intake
EHC	Enzyme hydrolyzed casein
EAAs	Essential amino acids
HDP	Highly digestible protein
IDE	Ileal digestible energy
IEAA	Ileal endogenous amino acid
KTI	Kunitz trypsin inhibitor
MBM	Meat and bone meal
N	Nitrogen
NEAAs	Non-essential amino acids
NSP	Non-starch polysaccharide
NFD	Nitrogen-free diet
SAA	Synthetic amino acids
SBM	Soybean meal
SCP	Single-cell protein
SIAAD	Standardized ileal amino acid digestibility
TID	True ileal digestibility
Tis	Trypsin Inhibitors
TLR	Toll-like receptor
IL	interleukin
SCFA	Short chain fatty acid
SIS	Sodium-iodine symporter
